# Caricatured facial movements enhance perception of emotional facial expressions

**DOI:** 10.1177/03010066221086452

**Published:** 2022-03-28

**Authors:** Nicholas Furl, Forida Begum, Francesca Pizzorni Ferrarese, Sarah Jans, Caroline Woolley, Justin Sulik

**Affiliations:** Royal Holloway, 3162 University of London, UK; Royal Holloway, 3162 University of London, UK; Cognition, Values & Behavior, Ludwig Maximilian University of Munich, Germany

**Keywords:** biological motion, categorization, emotion, face perception, models, motion, facial expression, caricature, face space, similarity

## Abstract

Although faces “in the wild” constantly undergo complicated movements, humans adeptly perceive facial identity and expression. Previous studies, focusing mainly on identity, used photographic caricature to show that distinctive form increases perceived dissimilarity. We tested whether distinctive facial *movements* showed similar effects, and we focussed on both perception of expression and identity*.* We caricatured the movements of an animated computer head, using physical motion metrics extracted from videos. We verified that these “ground truth” metrics showed the expected effects: Caricature increased physical dissimilarity between faces differing in expression and those differing in identity. Like the ground truth dissimilarity, participants’ dissimilarity perception was increased by caricature when faces differed in expression. We found these perceived dissimilarities to reflect the “representational geometry” of the ground truth. However, neither of these findings held for faces differing in identity. These findings replicated across two paradigms: pairwise ratings and multiarrangement. In a final study, motion caricature did not improve recognition memory for identity, whether manipulated at study or test. We report several forms of converging evidence for spatiotemporal caricature effects on dissimilarity perception of different expressions. However, more work needs to be done to discover what identity-specific movements can enhance face identification.

## Introduction

### Challenges of Dynamic Face Perception

How do we represent dynamic faces? How much or what kind of motion information is used when perceiving different emotional expressions or different facial identities? These might seem like elementary questions, as moving faces are what people (and their visual systems) encounter and interact with on an everyday basis. Nevertheless, we know much more about perceptual representations of static photographs than those of dynamic faces. The reasons for this limitation have been mostly practical. Databases of static photographs have been widely available for some time. It is relatively easy to measure their low-level features (e.g., spatial frequency, colour, etc.) and static photographs are relatively easy to manipulate (e.g., to morph and average). Also, facial identities and emotional expression apices are readily recognisable from static photographs.

Nevertheless, visual systems could plausibly benefit from representing facial dynamics in addition to static shape and texture information. Motion carries useful information of its own and, under some circumstances, motion can even facilitate extraction of static form (i.e., structure from motion) (O’Toole et al., 2002). Also, unpredictable dynamics should interfere with performance, as it is more difficult to reliably extract static form from a fleeting and unstable stimulus. Indeed, even the most sophisticated automated face recognition algorithms (e.g., O’Toole et al., 2018) remain largely limited to static image-based recognition. In contrast, humans actively use motion to perceive identity and expression (Lander & Butcher, 2020), as we discuss below. Consequently, previous authors have raised the question, still unanswered, about what is the specific content of these motion representations (Dobs et al., 2018). Below, we test a hypothesis, generalised from “face space” theories of static photograph perception (Valentine, 1991; Valentine et al., 2016), that *distinctive motion* enhances identity and expression perception.

Indeed, there is evidence that motion assists perception of famous and unfamiliar faces (Lander & Butcher, 2020) over and above the assistance provided solely by shape information from static photographs. Here, we mainly review studies that (like our present study) focus on non-rigid motion—motion that deforms shape, such as facial feature movement (e.g., expressions), as opposed to rigid, shape-preserving motion (e.g., whole head motion). For facial expressions, motion assists categorisation, especially under challenging contexts, such as when form information is degraded (Krumhuber et al., 2013). For identities, there is also evidence that non-rigid motion helps discrimination and matching of unfamiliar identities in the absence of reliable form cues (Dobs et al., 2017; Girges et al., 2015; Hill & Johnston, 2001) and naming of famous faces in videos (Knight & Johnston, 1997; Lander et al., 1999; Lander et al., 2001). Motion assists perception of identity, over and above the contribution of form information (Knappmeyer et al., 2003; Pilz et al., 2006; Thornton & Kourtzi, 2002). Unfamiliar faces that exhibit non-rigid movements when they are studied, including expression movements (Lander & Chuang, 2005), confer improved recognition memory for test faces that are static (Lander & Bruce, 2003) or moving (Lander & Davies, 2007; Butcher et al., 2011). Preliminary evidence also exists that chronic inability to recognise faces (developmental prosopagnosia) can be improved by studying faces in motion (Bennetts et al., 2015).

### Is Face Space Spatiotemporal?

How is this motion represented and what kind of motion is useful for perceiving identities and expressions (Dobs et al., 2018)? We tested here the hypothesis that humans use a spatiotemporal face space, which predicts that distinctive movements are especially advantageous for perception. “Face space” theory (Valentine, 1991) conceives of face representations as dissimilarity spaces, where features of faces serve as axes and distances between faces (vectors) represent their dissimilarity. The feature axes which humans use are not directly known but attempts have been made to speculate about their content by computing and interpreting feature axes (e.g., “Eigenfaces”) derived from computational models operating on static images (Turk & Pentland, 1991). The face space conception partially owes its prominence to its ability to predict effects of facial distinctiveness on human performance (Valentine, 1991). Faces in the space are often assumed to be distributed such that individual exemplar faces similar to the average (i.e., typical face exemplars) are situated in a more densely-populated area of the space (Burton & Vokey, 1998). This leads to the prediction that these typical faces should be more confusable with each other and therefore less accurately recognised, whereas more distinctive faces are better recognised.

Here, we propose an elaboration of the traditional face space conception. Because previous studies and models of face space to date relied on static photographs, they necessarily focused on “image-based” dimensions of face space, which are exclusively spatial and in nature based on facial form information. It is not clear how purely spatial face space dimensions could be used to encode motion information from faces. A truly spatiotemporal face space would need to be more of an inherently dynamic process where motion perception leads to projection of faces onto spatiotemporal dimensions, and where perception of static images is essentially a “snapshot” of this process. In the study, we created an animated stimulus set where the animations varied on motion-based dimensions. We then can test the extent to which participants’ face spaces incorporate the same variability along these motion-based dimensions.

We further test a hypothesis that participants implement a spatiotemporal face space that, like previously studied purely spatial face spaces, is susceptible to effects of caricaturing. Caricaturing is a technique that many previous studies of static facial photographs have used to show that humans implement a face space representation. Caricaturing involves the alteration of a veridical (original) face to make it more similar to a face average (an anticaricature) or more dissimilar to it (a caricature). Here, we applied a caricaturing technique to facial expression movements. Thus, in our study, *caricatures* are facial expression movements that exhibit exaggerated distinctiveness, relative to their veridical versions, because caricatures have been rendered at a location in a spatiotemporal face space more distant from the norm movement (i.e., the average or prototype movement). In the same vein, *anticaricatures* in the present study are facial expression movements that exhibit diminished distinctiveness and more closely resemble the norm than their original veridical versions, because anticaricatures have been rendered at a location in spatiotemporal face space nearer to the norm movement.

While many previous studies investigated caricatured, veridical, and anticaricatured faces, these studies have caricatured only the two-dimensional spatial information in photographs, rather than motion derived from video. The earliest of these spatial caricature studies relied on a technique for caricaturing line drawings (Brennan, 1985). Numerous studies since then have created photorealistic caricatures involving caricature of shape or texture information in image pixels (Benson & Perrett, 1991). Consistent with the prediction of face space theory, these studies demonstrated beneficial effects of degree of facial caricature (i.e., experimentally manipulated distinctiveness) on performance. In the context of expression categorisation (i.e., explicit labelling of expressions by name), basic expressions are learned in daily life in their veridical versions, and so caricatured expressions serve as “superportraits” of what has been learned (Rhodes, 1997). Considerable evidence shows that degree of caricature of basic expressions increases categorisation accuracy and perceived emotional intensity (Benson et al., 1999; Calder et al., 1997; Calder et al., 2000; Kumfor et al., 2011; Lane et al., 2019). In the context of identity processing, degree of caricature improves naming of famous (Frowd et al., 2012; Lee et al., 2000) and familiar (Frowd et al., 2007) faces and facilitates learning of the names of unfamiliar faces (Stevenage, 1995), including in cases where performance is notoriously inaccurate because of low-resolution faces, other-race faces or older participants (Dawel et al., 2019). Caricature also facilitates detection of probe identities within arrays of faces (McIntyre et al., 2013).

Caricature of static photographic images benefits perception because spatial information is rendered distinctive. Here, we caricatured spatiotemporal information from facial videos (i.e., the size, speed and timing of movements of facial features), instead of purely spatial information, to test the hypothesis that face space also incorporates such spatiotemporal dimensions. We then tested for several caricature effects on performance that are predicted by face space theory. The three studies we report here implemented paradigms that measure dissimilarity perception (Studies 1 and 2) or recognition memory for unfamiliar dynamic faces (Study 3). These paradigms, in addition to the ones discussed above, have been used to investigate 2D spatial caricature effects on perception, as we discussed in the next section.

### Caricature Effects on Perceived Dissimilarity of Expressions and Identities

Our Studies 1 and 2 test the hypothesis that findings from previous studies that measured dissimilarity perception of static image caricatures will generalise also to the case of spatiotemporal caricature. In such studies, participants view trials where two faces are presented simultaneously with different identities but at the same degree of caricature. Participants, then, perceive greater dissimilarity between the different identities, when they are presented at higher degrees of caricature (Dawel et al., 2019; Lee et al., 2000; Irons et al., 2014; 2017; Lane et al., 2018; McKone et al., 2018). Some studies of dissimilarity perception further used multidimensional scaling (MDS) to visualise this caricature-induced expansion of perceptual distances between identities in face space (Johnston et al., 1997; Lee et al., 2000). The interest of these previous studies was mainly in perception of different identities. Here, we also measured perceived dissimilarity between *expression categories*, in addition to between identities.

Our report of Study 1 below includes a review of an analysis that we previously reported in Furl et al. (2020). In this analysis, we used data from a pairwise similarity rating task to show that spatiotemporal caricature increases perceived dissimilarity for different expressions but not different identities. In the present manuscript, we probe this finding even further. We compare those results alongside those of a new study (Study 2), which used a multiarrangement task, instead of the more traditional pairwise ratings task, to measure dissimilarity perception (Kriegeskorte & Mur, 2012). Then, using datasets from both Studies 1 and 2, we implemented novel analysis methods to test for convergent evidence of caricature effects across the two studies on perceived dissimilarity of faces that differ in expressions.

The construction of our caricatures involved the computation of physical motion metrics (based on models fitted to video landmark tracking data), which profile the physical movements of each stimulus. This physical quantification of the motion of each stimulus might be considered “ground truth,” because it is an objective physical measurement from the stimulus, which quantifies the information afforded to participants for use during perception. In contrast, participants’ dissimilarity perception is subjective, may or may not incorporate information sampled from the ground truth, and depends on the encoding mechanisms and representations that participants naturally use. Having these ground truth profiles of physical motion metrics for each stimulus, we are in a position to implement new analyses that most studies of spatial caricature were not in a position to implement. Namely, we can directly compare physical ground truth dissimilarities between stimuli with human dissimilarity perception of these same stimuli. From this comparison, we can infer whether the objective (i.e., ground truth) and subjective (i.e., behavioural) face spaces are similarly structured—that is, do they share “representational geometry” (Nili et al., 2014)? This analysis would be crucial in the event that caricature does not enhance dissimilarity perception between pairs of different identities or pairs of different expressions. If our analysis of ground truth shows that caricature did successfully enhance identity-specific or expression-specific distinctiveness of the stimuli, then we must infer that participants’ encoding mechanisms were not sensitive to this information, even though it was available in the stimulus for their use.

### Caricature Effects on Recognition Memory for Identities

In another new study (Study 3), we implement an alternative method for testing whether participants’ representations incorporate identity-diagnostic spatiotemporal distinctiveness. In practice, humans rarely explicitly express perceived dissimilarity between pairs of faces. Such dissimilarity measures, therefore, might be treated as only indirect proxies for expression categorisation and identity recognition. The latter are judgements more typical of everyday human activities and might be thought of as “direct perception” of identity and expression. Moreover, dissimilarity-based paradigms can have a limitation, in that the exact instructions given to participants about how to perform their ratings can lead to demand characteristics (i.e., they may use information for their judgements that they wouldn’t naturally use except for the instructions). Indeed, as every paradigm has strengths and limitations, convergent evidence across complementary paradigms can bolster a stronger case, compared to evidence from one paradigm alone. In our previous work (Furl et al., 2020), we already went beyond dissimilarity perception and confirmed the hypothesis derived from face space theory that basic expressions are accurately categorised by expression label when caricatured than anticaricatured. Here, we adapt a similar approach, except to study spatiotemporal caricature effects on perception of unfamiliar identity. Specifically, we measured participants’ ability to discriminate previously studied identities from newly presented identities (i.e., “recognition memory”).

A considerable number of studies using static images suggest that increased distinctiveness enhances recognition memory for facial identity. Natural facial distinctiveness improves recognition memory for unfamiliar faces (Hancock et al., 1996; Light et al., 1979). And the degree of static image caricature improves recognition memory of studied unfamiliar faces when the same caricature degree is applied at both study and test (Irons et al., 2014; 2017; Itz et al., 2014; Kaufmann et al., 2013; Kaufmann & Schweinberger, 2012; Schulz et al., 2012; Schultz et al., 2012) and when caricature of new (non-studied) faces is manipulated at test (Deffenbacher et al., 2000; Irons et al., 2014; 2017). We focused in Study 3 on two effects, which others have termed “generalisation” (Itz et al., 2017) and “superportrait” effects (Table 1).

**Table 1.** Stimulus caricature levels used in Study 3.

Caricature effect	Faces at study	Faces at test
Generalisation	Caricatured or anticaricatured	All veridical
Superportrait (hits)	Veridical	Caricatured, anticaricatured
Superportrait (false alarms)	N/A	Caricatured, anticaricatured

The first of these two effects, generalisation (Table 1), has been demonstrated by studies that show that degree of caricature of unfamiliar faces when they are studied enhances their recognition when they are later tested in their veridical versions (Deffenbacher et al., 2000; Itz et al., 2017), although this hasn’t always replicated (Rodriguez et al., 2008; Rodriguez & Gutierrez-Osuna, 2011). There is already some suggestive evidence that generalisation effects might arise for spatiotemporal caricature: Natural motion distinctiveness of study faces enhances recognition memory of static test faces (Lander & Chuang, 2005).

We also tested in Study 3 whether spatiotemporal caricature of unfamiliar faces produces a second effect on recognition memory: a superportrait effect. One might expect, based on face space theory, that a face learned in its veridical version should be better recognised if presented at a higher degree of caricature at test, as the caricatured version is designed to be “more representative” of the identity than even the veridical face (Rhodes, 1997). Indeed, famous faces (learned in their veridical versions outside the laboratory) are named more accurately when caricatured (Frowd et al., 2012; Lee et al., 2000). The current empirical evidence for a superportrait effect appears relatively weak for the case of static photographs of unfamiliar faces. Nevertheless, we tested in Study 3 whether a superportrait effect might hold instead for the case of spatiotemporal caricature of unfamiliar dynamic faces. We incorporated features from previous experimental designs (Deffenbacher et al., 2000; Kaufmann & Schweinberger, 2012), which included conditions where faces were studied in their veridical versions and then degree of caricature was manipulated at test (Table 1).

### Using Spatiotemporal Caricature to Study Face Space

Previous authors (Dobs et al., 2018) proposed that computer-animated faces would be advantageous for experimentally manipulating facial movements. Our spatiotemporal caricature technique resembles these methods, as established by others (Dobs et al., 2014; Hill & Johnston, 2001; Kätsyri & Sams, 2008). Landmarks are tracked from the movements of human facial features and their time courses quantified (often via curve fitting using nonlinear functions). Then, once the time courses have been manipulated as needed, they are used to animate a computerised head model or avatar. Because the movements of the animations are already quantified in advance, this method affords comparison of the physical ground truth dissimilarity of the stimulus motion metrics with participants’ perceived dissimilarity.

We validated our spatiotemporal caricature technique in a previous study, which showed that the computer-animated expressions were not any less convincing than the original expression videos from which they were derived. Moreover, movements with higher caricature degree had expressions that were, as expected, more convincing and more accurately categorised (Furl et al., 2020). These caricature effects resemble caricature effects on emotional intensity reported for point light displays of facial expressions (Pollick et al., 2003) and emotional speech movements (Hill et al., 2005).

Using these validated stimuli, we tested in the present study the hypotheses of face space theory that higher degrees of spatiotemporal caricature (distance from the norm movement) should: (1) increase ground truth dissimilarity in the physical movements of the face stimuli themselves between identities and expressions; (2) increase participants’ perceived dissimilarity between differing identities and expressions (Studies 1 and 2); (3) lead to a generalisation effect on recognition memory (Study 3); and (4) lead to a superportrait effect on recognition memory (Study 3).

## Methods: Spatiotemporal Caricature

Our previous publication describes in detail the creation of the dynamic face animations and their validation study (Furl et al., 2020). We summarise the most relevant content here. We derived the animated spatiotemporal caricatures from 2 s videos (50 frames), taken from the BU-4DFE video set (Yin et al., 2008), which show humans transitioning from a neutral expression to poses of apical emotional expressions. We selected six movements corresponding to conventional basic expression categories (anger, disgust, fear, happy, sad, surprise) for six female and six male identities.

We used an established automated landmarking tracking algorithm in the Psychomorph software (Chen & Tiddeman, 2010) to track from frame to frame 141 landmarks distributed throughout each face. Automated landmarking has been found to produce beneficial effects of 2D image caricature on identity perception even using only roughly half our number of landmarks (McKone et al., 2018). We previously reported magnetoencephalographic responses to this tracked motion for some of the identities used here (Furl et al., 2017). For purposes of animating our computerised head model, we selected 15 “key feature” landmarks. These key features correspond to the parts of the face with the most degrees of freedom for non-rigid motion and are thought to influence perception of basic expression categories (Delis et al., 2016) and map directly onto the moveable parts of the animated head model that we used (e.g., eyelids, eyebrows, corners of mouth, etc.).

**Figure 1.**
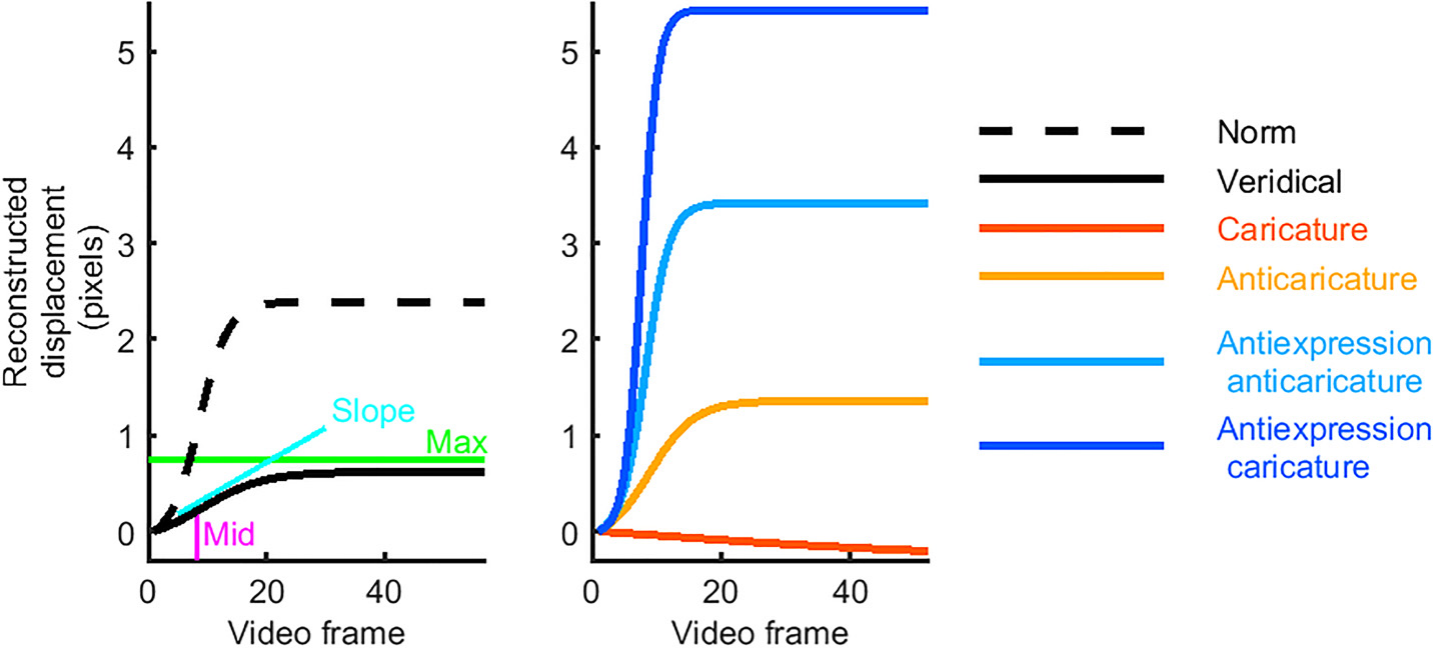
Sample movement time courses. The left plot shows timecourses that result from fitting the three parameters (slope, mid, and max) of logistic models to movement data measured in video. The three parameters are illustrated using a sample timecourse (black solid line), fitted to an eyebrow raise from one individual's surprise expression. Also shown is the “norm” timecourse (dashed line), fitted to the average across all movements of this feature. This veridical timecourse has a slower slope, a later mid and a lower max than the norm for this feature. The right plot shows the new, artificial timecourses, which served as bases for our caricatured stimuli. The reconstructed caricature timecourse is slower, later and has a lower max than the veridical timecourse. The antiexpression caricature, however, is the opposite: faster slope, earlier mid, and higher max.

We computed physical metrics of motion at each key landmark in each video by fitting logistic functions to the pixel-displacement timecourses. Indeed, the nonlinear nature of movement timecourses has been shown to be important for expression recognition (Korolkova, 2018). When fitting, we fixed the starting position parameter to zero displacement and estimated the remaining three free parameters of the logistic function. These parameters we could then use as motion metrics, which characterise the motion in each of the videos. The *maximum metric (“max”)* is the size of the movement; the total number of pixels displaced. The *slope metric* can be interpreted as the speed of the movement. The *midpoint metric (“mid”)* is the time point centred on the displacement and can be considered how early or late the movement took place. Figure 1 schematises these metrics.

Although we made an effort to select a relatively convincing face set and to choose the most convincing examples of expression movements from it, the selected expression exemplars still varied widely in how convincing their expression poses appeared, like most facial expression videos sets available at the time. Our first attempts at animations based on raw motion data extracted directly from the videos yielded (according to our subjective standards) poorly realised expressions and unnatural-appearing identity-specific movement. Thus, to produce animations with more convincing expressions and better-controlled identity-diagnostic movements, we modified the motion information extracted from individual exemplar videos. Our approach involved mathematically modelling identity-specific movements as exemplars normally distributed around an averaged category prototype. To realise this model, we recomputed each motion metric in every video to equal its *expression prototype;* that is, the average of the motion metric for that feature over 11 of the identities showing the same expression. We then added to each feature's motion metric some *identity-specific, characteristic motion*, which was a value sampled from a Gaussian distribution, with a mean equal to the expression prototype value for that metric for that feature and with its SD scaled to 20% of its mean value. The results of our published validation study (Furl et al., 2020) show that this approach successfully produced basic expressions that were convincing to participants.

This statistical model of normally distributed variability around average movement values is distinct from one that uses pure (i.e., white) noise, in which any movement could have been sampled with uniform probability. We did not expect *a priori* that the simple addition of noise would produce biologically plausible expression movements, nor had we any *a priori* reason to suspect that natural identity-specific movements would be distributed uniformly. Instead, we sought a model of individual variability that would be biologically plausible and would conform to theoretical cognitive models of how participants learn new concepts and visually recognise exemplars. We therefore rooted our model of identity-specific motion within a classical tradition: normally distributed variability around a norm or prototype value. Normal distributions around an average value are well known to naturally arise in biology for measures ranging from height to intelligence quotient. Indeed, even routine parametric statistical tests make similar assumptions about distributions of samples from populations. Common signal detection models (e.g., the computation of *d′*) assume normally distributed variability of individually experienced items (Macmillan & Creelman, 1991). In empirical paradigms in psychology, categories, and concepts have historically been manipulated experimentally as variability centred on an average (e.g., Posner & Keele, 1968). Common visual recognition models such as Gaussian general recognition theory (Ashby & Townsend, 1986) and norm-based face space theory and similar models (Ross et al., 2014) are fundamentally rooted in the idea that distributions of category exemplars approximate Gaussian distributions, with an expectation of greater exemplar density near the average value (Burton & Vokey, 1998). Our approach to modelling identity-specific exemplars of expression categories builds on this tradition.

At present, identity-specific expression movements have not been studied at sufficient empirical detail to assert with confidence *a priori* exactly how natural expression movements might vary over individuals. Moreover, there are few studies of human perception that might serve as an *a priori* guide to which types of identity-related movement information the human visual system might be sensitive. We present our approach as an early attempt to test hypotheses about identity- and expression-specific information to which the visual system might hypothetically be sensitive. Here, we attempted to exaggerate, relative to the average movement, the distinctiveness of both these identity-specific movements and the prototypical expression movements. Later, in the Results, our ground truth analysis will verify indeed that caricature successfully enhanced dissimilarity of our motion metrics both for different expression categories and for different identities. Because we have carefully quantified and controlled the expression and identity-specific information in our face set, we will be able to draw conclusions about to exactly what kinds of distinctive information about expressions (prototypic) and identities (normally variability around expression prototype) the visual system is (or is not) sensitive.

Before animating our head model, caricature proceeded by separately contrasting each (modified) motion metric (max, slope, mid) for every key landmark in every video with the average metric over 66 of the face videos. That is, we compared each individual's motion parameters (as defined above) to those of the average movement (averaged across all identities and expressions) and then exaggerated these individual parameters relative to the average to make caricatures (or similarly altered them to create our different caricature levels). This number of averaged exemplars compares favourably to averages used in previous studies of static image caricature, as reviewed by Furl et al. (2020). We attempted to maintain consistency with the terminology of previous literature (Blantz et al., 2000; Calder et al., 2000), using the following nomenclature for stimuli used across the three studies we report here:

- “Caricatures” (dissimilarity with average multiplied by 1.7)
- “Veridical expressions” (dissimilarity with average multiplied by 1)
- “Anticaricatures” (dissimilarity with average multiplied by 0.85)
- “Antiexpression anticaricatures” (dissimilarity with average multiplied by −0.85)
- “Veridical antiexpressions” (dissimilarity with average multiplied by −1).
- “Antiexpression caricatures” (dissimilarity with average multiplied by −1.7)

Antiexpressions, once animated, do not appear as basic emotional expressions but as novel “expressions” whose key features have the opposite distinctiveness as their corresponding basic expressions. Presumably, participants would not easily perceive anti-expressions in terms of the original basic expression categories.

Using the computer graphics software Blender, we animated the key features of a head model based on new logistic timecourses, which we computed from the caricatured metrics. We gave the head model the shape-normalised appearance of individuals from the videos by using the full set of landmarks to register the pixel maps from video frames to corresponding landmark points on the surface of the head model. Caricatured videos are openly available at https://openneuro.org/datasets/ds002741/versions/1.0.2.

## Methods: Studies 1 and 2

Links to on-line demonstrations of Studies 1 and 2 can be found at https://osf.io/ft53e/.

### Participants

Study 1 (Furl et al., 2020) enrolled 592 participants (313 females, 276 males, three self-described or opted-out) and Study 2 enrolled 533 participants (250 females, 276 males, 7 self-described or opted out). Participants accessed the study online via Amazon Mechanical Turk and were screened to be from the United States with 95% approval ratings on the platform. Ethics protocols were approved by the Royal Holloway, University of London College Ethics Board.

### Stimuli

Study 1 used 180 face videos = nine identities (four female) × five expressions (anger, disgust, fear, happy, surprise) × four caricature levels (caricature, anticaricatures, antiexpression caricatures, antiexpression anticaricature). Study 2 added a tenth identity (female), resulting in 200 total videos.

### Procedures

**Figure 2.**
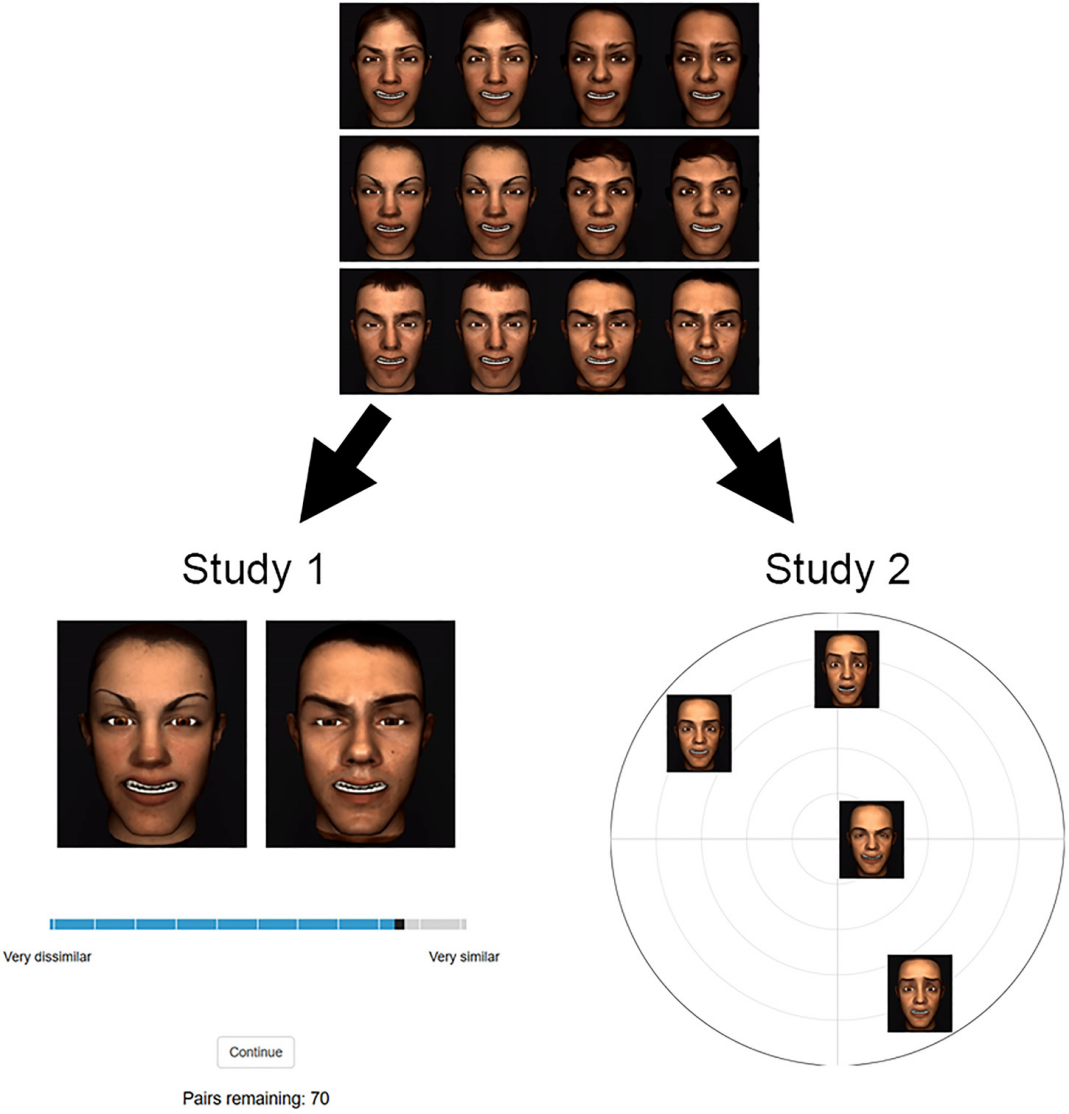
Example trials from Studies 1 and 2. Participants were first familiarised with a set of stimuli to which they would be responding. At the top is an example set with the same expression, but identities can differ. The lower left shows an example of a Study 1 trial, in which a participant rates the similarity of a pair of faces with the same expression but different identities. The lower right shows an example of a Study 2 trial where faces have the same identity, but expressions can differ. In Study 2, participant arranged faces within these arenas by dissimilarity.

Both studies were programmed in jsPsych (De Leeuw, 2015). The studies aimed to characterise how motion caricature affects participants’ perceptual face space by measuring perceived dissimilarity between pairs of faces with different expressions or identities. These studies sought convergent, replicated evidence across two separate tasks (Figure 2). In Study 1, participants provided similarity ratings between pairs of animations. Study 2 used a spatial arrangement paradigm to obtain perceived dissimilarities between face videos.

**Figure 3.**
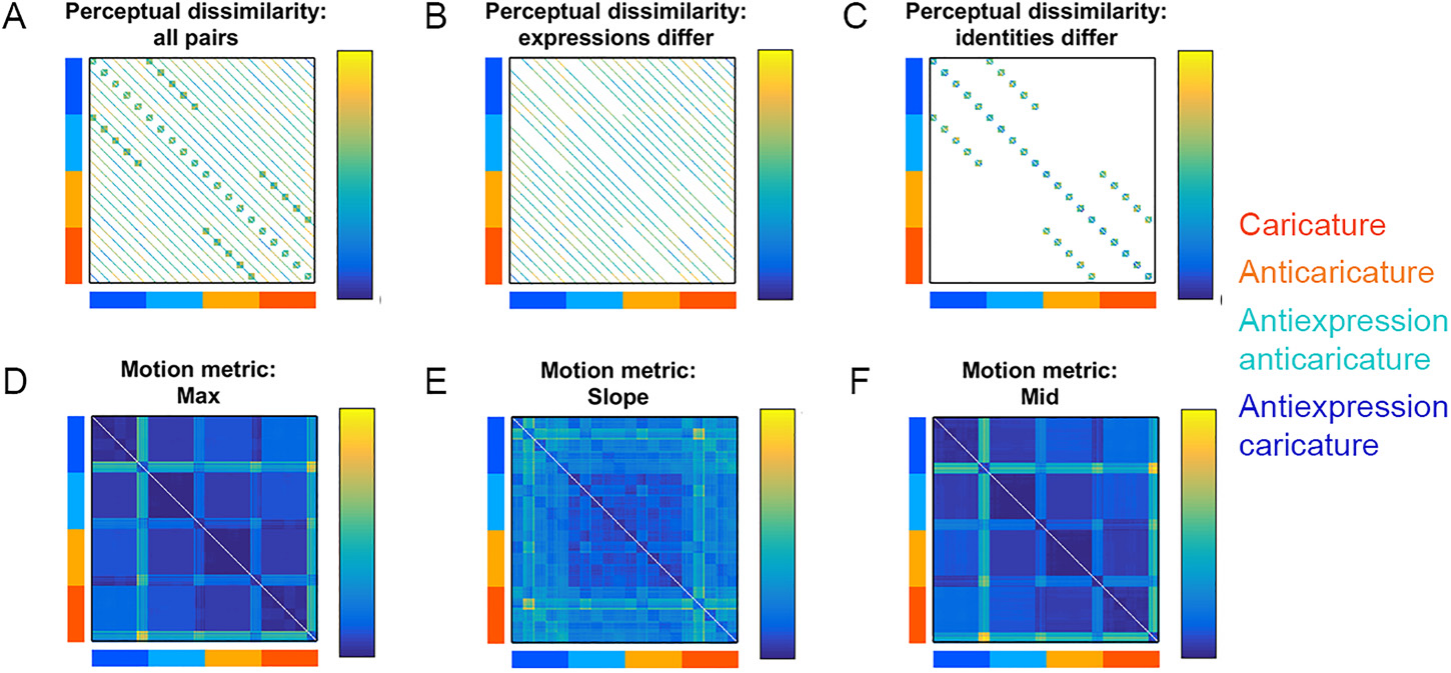
Dissimilarity matrices for the 200 videos used in Study 2. Caricature conditions of rows and columns are colour-coded according to legend. White cells contain no data. Participants’ perceptual dissimilarity is shown for (A) all video pairs, (B) pairs where expressions differ or (C) pairs where identities differ. Dissimilarity data (Euclidean distances) between feature patterns of physical motion metrics are shown for max (D), slope (E), and mid (F).

Figure 3A shows the 200  ×  200 “grand matrix” for Study 2, whose values are symmetric around the diagonal and whose cells could, in principle, hold dissimilarity values for all possible pairs of the 200 videos (see *Stimuli* section). In Studies 1 and 2, we filled in sections of this dissimilarity grand matrix using participants’ perceived dissimilarity (Figure 3A left, “all pairs”). Studies 1 and 2 specifically populated those cells where the expressions of the pairs are different (“expressions differ,” Figure 3A, middle) and cells where the identities of the pairs are different (“identities differ,” Figure 3A right). We focused data collection on these specific sections of the grand matrices so that, during data collection, we could manage the combinatorial explosion of face pairs, keep the study sessions to a comfortable duration per participant and still obtain data to directly examine caricature effects on dissimilarity between different identities and expressions.

To populate these cells for Studies 1 and 2, we assigned each participant to one of two between-participants conditions, which were defined by the face sets with which the participants engaged. In the first condition (283 participants in Study 1 and 212 participants in Study 2), for a given participant, faces exhibited all four caricature levels from five basic expressions, but all faces were the same identity (randomly selected for each participant out of the total number of identities). An example of a Study 2 trial from this condition is shown in the lower right of Figure 2. In the second condition (309 participants in Study 1 and 321 participants in Study 2), the faces included caricatures and anticaricatures of six of the identities but were all the same expression or antiexpression (randomly selected from the 10 expressions plus antiexpressions available). An example of such a subset of faces from this condition is shown at the top of Figure 2 and an example Study 1 trial from this condition is shown at the lower left.

In both studies, before providing any behavioural data, each participant first studied the face set assigned for that session (videos played in a grid on a continuous loop) for as long as desired (Figure 2, top). In both studies, we explicitly instructed participants when making their dissimilarity decisions to account for the faces’ movements, whether or not videos were from the same category (i.e., identity or expression category).

In Study 1 (Figure 2, lower left), participants viewed simultaneously-presented pairs of faces side by side and rated them on a 100 point scale using a sliding scale spanning “Very dissimilar” (on the left) to “Very similar” (on the right), with the initial slide position set to 50. Trials were self-paced and videos played in a continuous loop. Four attention checks per participant appeared in the form of a heavily-pixelated animation, and participants were asked to respond “Very similar” every time this animation appeared. Participants were relatively attentive (82% of participants accurately responded to at least two animations) and the person-total correlation was high (mean *r* = .56) so we retained the full dataset for analysis.

On each trial of Study 2 (Figure 2, lower right), participants viewed small versions of the videos (playing in a continuous loop), initially placed outside of a circular arena. Participants then used a mouse to drag the videos into the arena and re-arrange their spatial locations within the arena so that videos that they perceived as more similar would be nearer to each other and videos that they perceive as more dissimilar would be further away from each other (Goldstone, 1994). The first such trial included all videos in the set designated for that participant and then a “lift the weakest” algorithm successively created new trials, with new arenas, each containing a different subset of the videos designated for that participant (see next paragraph for algorithmic details). As the arenas progress, the algorithm cumulatively optimises the amount of evidence gathered for the dissimilarity values between every stimulus pair. The goal is for all pairs of stimuli to eventually garner roughly equal amount of evidence. The algorithm can be stopped at any chosen quantity of evidence. Our study timed out at 10 min, but finished sooner if the evidence weight thresholds for all items were >0.5. Inverse MDS (Kriegeskorte & Mur, 2012; Richie et al., 2020) recovered the dissimilarity values from each participant. We did not implement attention checks in Study 2.

Mathematical details of the list the weakest algorithm can be found in the Appendix of Kriegeskorte and Mur (2012). In brief, the algorithm acquires its name because it populates each new area with the stimuli that currently have the weakest evidence for their dissimilarities. These evidence values are computed before each new arena from an on-the-fly computation of the dissimilarity matrix using inverse MDS. Higher on-screen distances are assumed to reflect higher signal to noise measurements and to add better evidence, with very short on-screen distances possibly more contaminated by random placement error. Thus, new arenas can be thought of as attempts to “zoom in” on excessively (relatively) densely-packed areas of the dissimilarity matrix. Using these evidence values, the algorithm populates each new arena using a heuristic greedy search to locally maximise “trial efficiency”—a ratio of the utility of the new evidence gained to the time cost (a function of the number of stimuli in the new arena). That is, the arena is initially populated only with the stimulus pair with the weakest evidence and then the next weakest stimuli are successively added. The final set of stimuli is chosen for the next arena when adding new stimuli to the set fails to further increase the trial efficiency.

## Results: Studies 1 and 2

We predicted, first, that degree of spatiotemporal caricature would enhance both physical (ground truth) dissimilarity (i.e., motion metrics) and participants’ perceived dissimilarity in both studies. That is, when the degree of motion caricature is increased, faces with different identities or different expressions should become more distinct from each other (distant from each other in face space) than their anti-caricatured counterparts. These anticaricatures, by contrast, should be more similar to each other and closely clustered in face space (Lee et al., 2000). This pattern of results should hold for physical stimuli (motion metrics), but the participants’ perceived dissimilarities are expected to correspond to this pattern also. This analysis therefore involves examining the mean distances among all stimuli that are caricatured (including expressions and antiexpressions) and testing whether these are larger than the mean distances among all stimuli that are anticaricatured (including expressions and antiexpressions). Our datasets enabled us to separately test whether this caricature-related expansion affects motion information that distinguishes different expressions (expressions differ) and motion information that distinguishes different identities (identities differ).

### Caricature Effects on Ground Truth Dissimilarity

**Figure 4.**
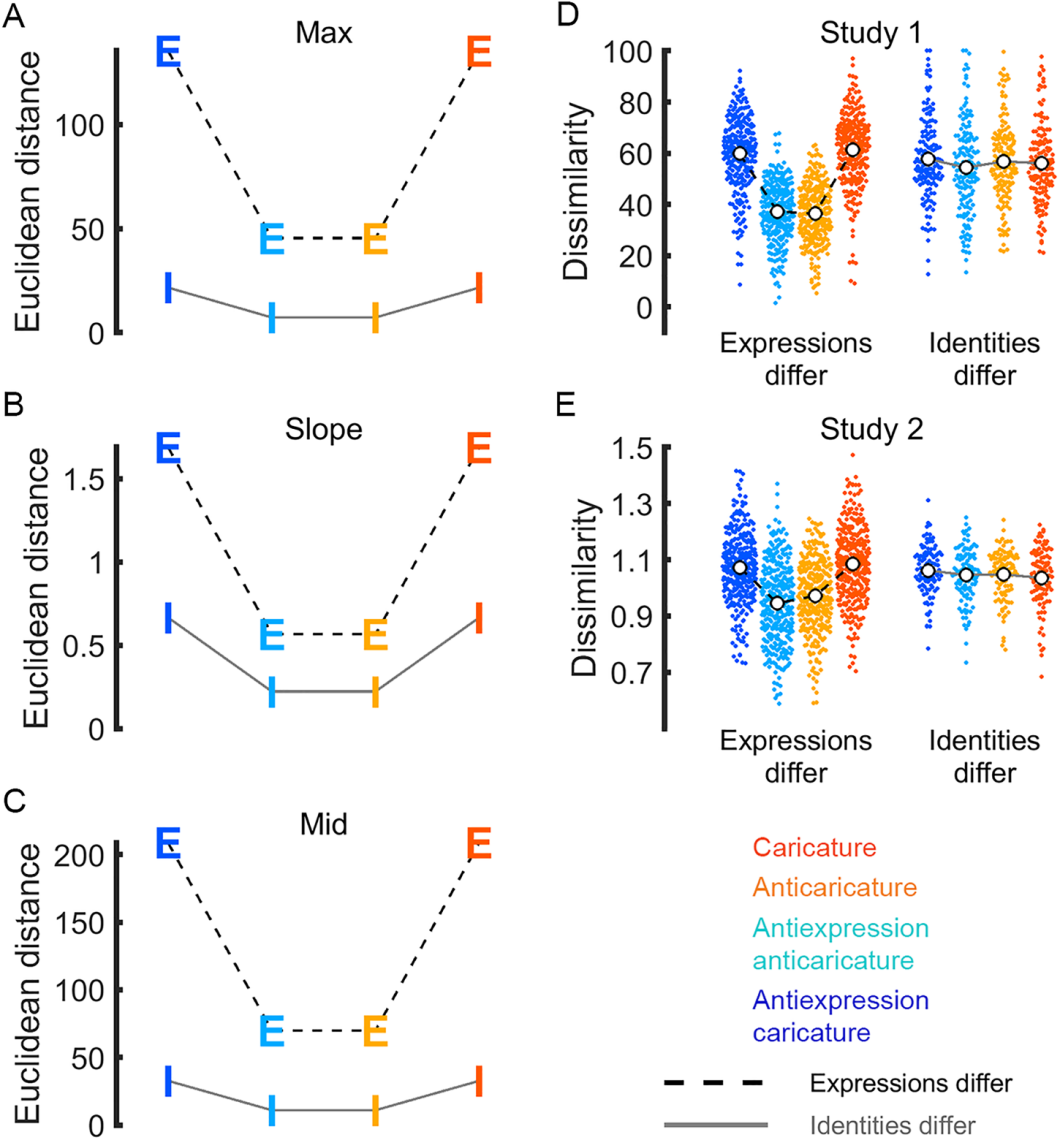
Ground truth dissimilarity of same-caricature-level pairs for motion metrics max (A), slope (B), and mid (C). Letters are coloured by caricature level, as in legend. Caricatures are physically more dissimilar than anticaricatures, both when pairs differ in expression (dashed line, letter E) and when pairs differ in identity (grey line, letter I). Perceived dissimilarity of same-caricature-level pairs is shown for Studies 1 (D) and 2 (E). Points for individual participant means are coloured by caricature condition, as in legend. White dots show caricature condition means.

Motion information that distinguishes different identities or expressions must be physically available in the stimuli in the first place if participants are also going to perceive this dissimilarity. Because we constructed our animations by manipulating motion metrics, we can use these same motion metrics to test whether caricature degree in fact makes movements more physically distinguishable. For each motion metric (max, slope, mid), we took Euclidean distances between the feature vectors for every pair of faces to populate grand dissimilarity matrices (Figure 3D to F). Figure 4A to C shows the average distance among caricatures and anticaricatures separately for face pairs that differ in expression/antiexpression category (“Expressions-differ” pairs, labelled “E”) and for face pairs that differ in their identities (“Identities-differ” pairs, labelled “I”). As expected, Euclidean distances between caricatured pairs were longer than distances between anticaricatured pairs for all three metrics, whether or not the pairs differed in identity or expression. This caricature effect was noticeably larger for pairs that differ in expression than pairs that differ in identity, suggesting that the movements of different expressions are more distinct than those of identities. Note, however, that the large values for expression dissimilarities were driven primarily by a few surprise expressions with unusually large (outlying) logistic parameter values. In sum, whatever movements make different identities distinct and whatever movements make different expressions distinct were both successfully enhanced by our caricature technique. This information was therefore available in the dissimilarity structure of the physical stimuli to be potentially perceived by the participants, if they are receptive to it.

### Caricature Effects on Perceived Dissimilarity

Below, we will show that, in Studies 1 and 2, participants’ perception does not exactly match the ground truth shown in Figure 4A to C and described in the preceding section. Participants appear to be only receptive to the distinctiveness of physical motion information that distinguishes different expressions, while they are insensitive to distinctive information that distinguishes different identities in our stimulus set. As with the analyses of motion metrics reported in the previous section, we compare the mean perceptual distance in face space among caricatures to the mean perceptual distance among anticaricatures. To amass evidence for our claim, we compare side-by-side the parallel results of our new analyses of motion metrics (described in the previous section and plotted in Figure 4A to C), our previously reported (Furl et al., 2020) analysis of pairwise ratings data in Study 1 (replotted in Figure 4D) and new results from a very different task (multiarrangement) in Study 2 (Figure 4E). In Studies 1 and 2, caricature appears to increase the mean perceived dissimilarity, relative to anticaricature. However, this pattern is visible only for face pairs where expressions differ. This pattern holds whether the faces exhibit basic expressions (in orange and red) or antiexpressions (In cyan and blue).

We confirmed this pattern statistically using frequentist linear mixed models in the software JASP (version 0.14; JASP Team, 2020), including fixed effects factors for condition (identity or expressions differ) and caricature level (all four levels) and a random effects grouping factor with random slopes by participant. Furl et al. (2020) previously reported an ANOVA-based analysis of these Study 1 data and here, we upgrade this analysis by using instead the linear mixed model approach. In this new analysis of Study 1 data, as expected, the condition  ×  caricature level interaction *F*(3,656.42) = 206.56, *p* < .001 and main effects of condition *F*(3,582.28) = 49.22, *p* < .001 and caricature *F*(3,656.42) = 247.92, *p* < .001 were all significant. Likewise, in Study 2, the condition  ×  caricature level interaction *F*(3,536.07) = 22.77, *p* < .001 and main effects of condition *F*(1,697.08) = 22.82, *p* < 0.001 and caricature *F*(3,536.07) = 22.06, *p* < 0.001 were all significant. In both studies, post-hoc *t*-tests verified that the only significant differences between caricature levels were in the expressions differ condition, where participants perceived caricature and antiexpression caricature pairs to be more dissimilar than anticaricature and antiexpression anticaricature pairs (*p* < .05, Bonferroni corrected for 12 tests). In short, our new results from the linear mixed models from both Studies 1 and 2 converged on one interpretation: the degree of caricature expanded the physical distances between motion metrics (Figure 4A to C), whereas participants’ perception in both studies reflected this expansion only when faces exhibited different expressions or antiexpressions. We found less evidence for any sensitivity to caricature level when the faces in our stimulus set were different identities.

### Visualisations of Caricature Effects in Face Space

We followed methods from previous work on static image caricature (Johnston et al., 1997; Lee et al., 2000) by using MDS to compute average locations in face space of (a) expression and antiexpression categories and (b) identities. This way, we could visualise how caricature changes their dissimilarity structure, both for stimulus motion metrics and for participants’ perception.

**Figure 5.**
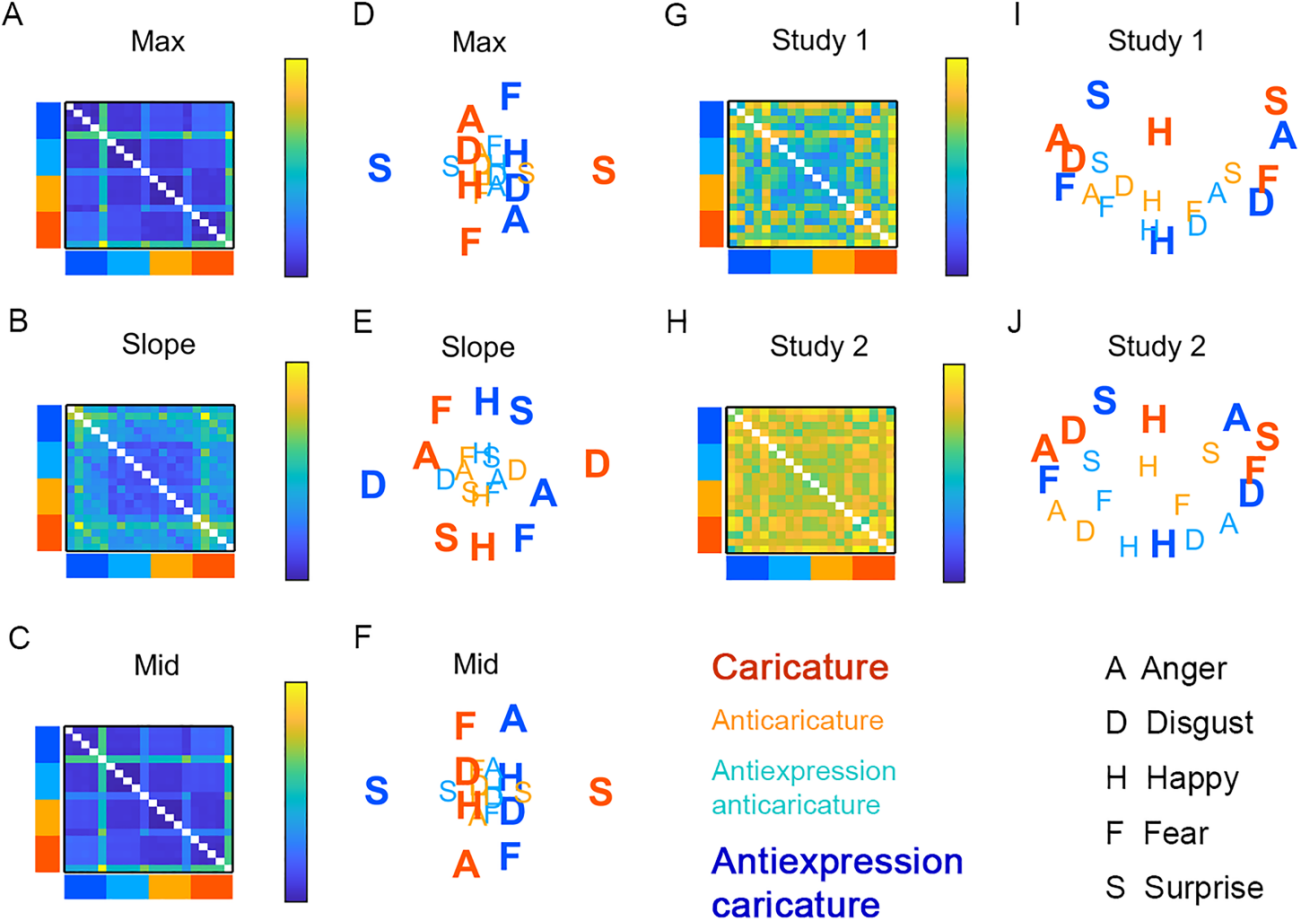
Expression/antiexpression dissimilarity, collapsing over identity. Matrices show ground truth dissimilarity for motion metrics max (A), slope (B), and mid (C) with multidimensional scaling (MDS) plots for max (D), slope (E), and mid (F). Other matrices show perceived dissimilarity for Studies 1 (G) and 2 (H), with MDS plots for Studies 1 (I) and 2 (J). Caricature levels of rows and columns in dissimilarity matrices and of letters in MDS plots are coloured as in the legend. Caricatures are shown in bold. A = anger, D = disgust, F = fear, H = happy, S = surprise. Caricatures appear more spread out in space compared to anticaricatures.

For all three motion metrics and human perception, we visualised the distances in face space between individual expression/antiexpression categories using two steps. The first step involved computing the average dissimilarity between all pairs of expressions/antiexpressions (collapsing over identities). We display in Figure 5 these recomputed dissimilarity matrices with 4 caricature levels × 5 basic expressions = 20 rows and columns for motion metrics max (Figure 5A), slope (Figure 5B), mid (Figure 5C), and the same matrices for participants’ perceived dissimilarity for Study 1 (Figure 5G) and Study 2 (Figure 5H). In the second step, we projected these expression category-specific dissimilarities onto a 2-dimensional plane using the best-fitting metricstress MDS solutions from 5000 random starting values (mdscale.m in MATLAB R2015B, The Mathworks, Nattick, MA) for motion metrics max (Figure 5D), slope (Figure 5E), mid (Figure 5F) and participants’ perceived dissimilarity for Study 1 (Figure 5I) and Study 2 (Figure 5J). An MDS analysis of expression categories using Study 1 data was previously reported in Furl et al. (2020). Here, we compare MDS of Study 1 alongside that of participants’ behaviour in Study 2 and those of the motion metrics.

MDS solutions based on motion metrics and participants’ behaviour in Studies 1 and 2 resemble each other. Caricatured expressions and antiexpressions (large bold letters) appear more spaced out and surround anticaricatures (smaller, lighter letters), which cluster towards the centres of the plots. This pattern is clearest for slope (Figure 5E) and, for all other plots, is most prominent for the first MDS dimension (*x*-axis). This “spacing out” of caricatured expressions provides another view on the caricature effects for “expression-differ” pairs shown in Figure 4D and E. Antiexpressions tend to occupy opposite sides of face space from their corresponding expressions. For example, surprise and antisurprise occupy opposite sides of the *x-* or *y*-axis. This we expected, as expressions and antiexpressions were designed to be distinctive in opposite ways of each other.

**Figure 6.**
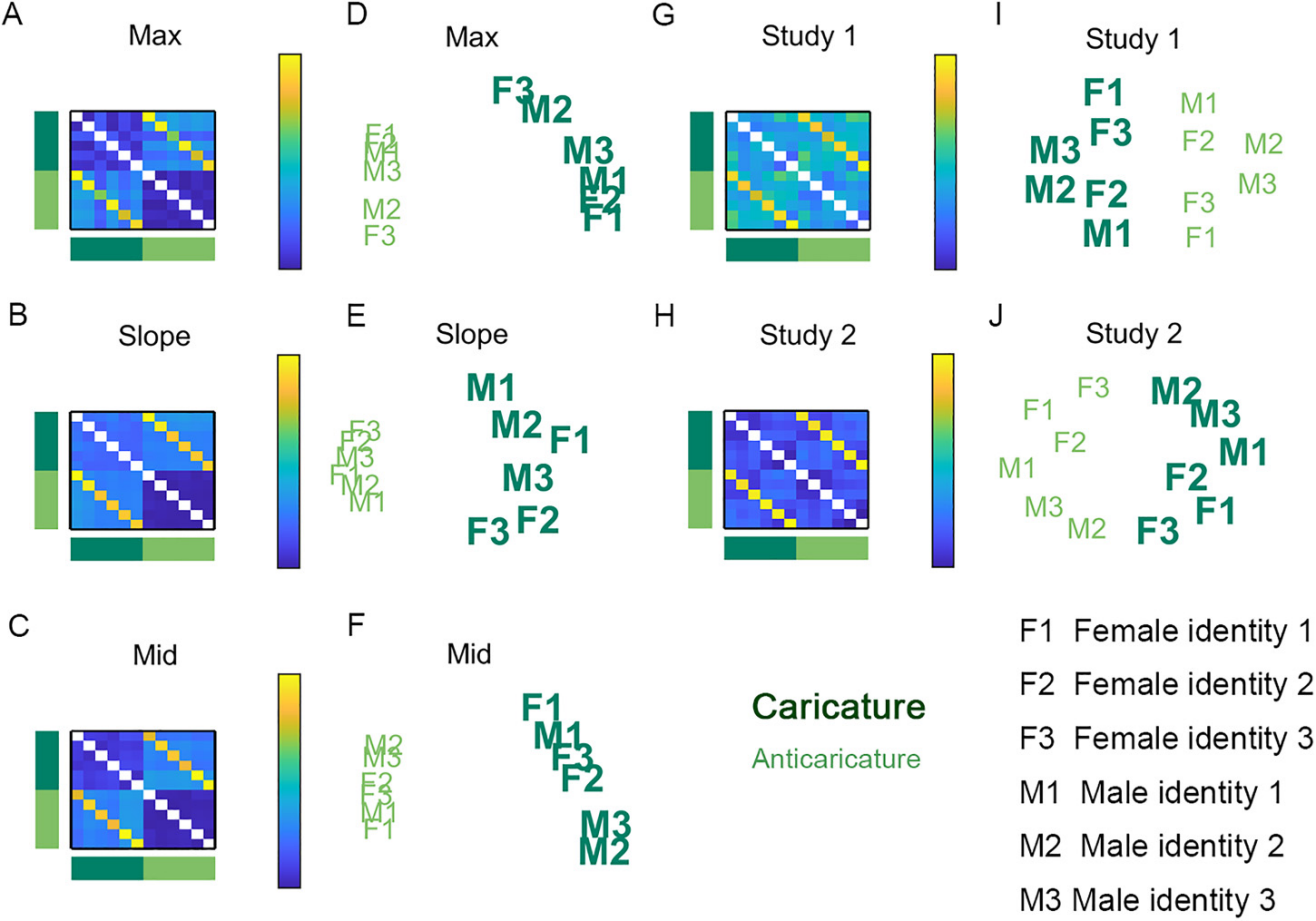
Identity dissimilarity, collapsing over expressions and antiexpressions. Matrices show identity dissimilarity for motion metrics max (A), slope (B), and mid (C). Also shown are corresponding multidimensional scaling (MDS) projections for max (D), slope (E), and mid (F). Also in matrices is perceived identity dissimilarity for Studies 1 (G) and 2 (H), with corresponding MDS projections for Studies 1 (I) and 2 (J). Dark green signifies caricatures and light green signifies anticaricatures. F1, F2, and F3 are female identities. M1, M2, and M3 are male identities. Caricature spreads out the physical movements of different identities compared to anticaricature but does not enhance perceived dissimilarity.

In an analysis that is new for both Study 1 and 2, we used a comparable method to visualise distances between individual identities (instead of expression categories) for all three motion metrics and for human perception. This time, in the first step, we averaged the dissimilarity between all pairs of the six identities (averaging over all expressions and antiexpressions). Note that, in our study design, all different identity pairs shared the same expression and so there were never pairs that could involve an expression paired with an antiexpression and so we were unable to consider pairs like these. We display in Figure 6 these recomputed dissimilarity matrices with 6 identities × 2 caricature levels (collapsing over expressions and antiexpressions) = 12 rows and columns for motion metrics max (Figure 6A), slope (Figure 6B), mid (Figure 6C) and the same matrices for participants’ perceived dissimilarity for Study 1 (Figure 6G) and Study 2 (Figure 6H) and MDS solutions for motion metrics max (Figure 6D), slope (Figure 6E), mid (Figure 6F) and MDS solutions for participants’ perceived dissimilarity for Study 1 (Figure 6I) and Study 2 (Figure 6J).

In the case of identities, the dissimilarity structure found for the motion metrics diverged somewhat from that of participant's perception. The first (*x*-axis) dimension of all plots clearly separates caricatures from anticaricatures. However, for physical motion metrics (Figure 6D to F), the caricatured identities (large bold letters) spread themselves out along the *y*-axis more than the anticaricatured identities (smaller, lighter letters), which are more clustered. This illustrates the findings reported in Figure 4A to C, where caricature degree leads to longer Euclidean distances between pairs of faces with different identities for all three motion metrics. In contrast, this expansion of distances by caricature appears absent from the MDS on the human participant data in Studies 1 and 2 (Figure 6I to J). This absence of expansion provides another view on the null findings for “identity-differ” pairs shown in Figure 4D and E.

### Representational Similarity Analysis (RSA)

**Figure 7.**
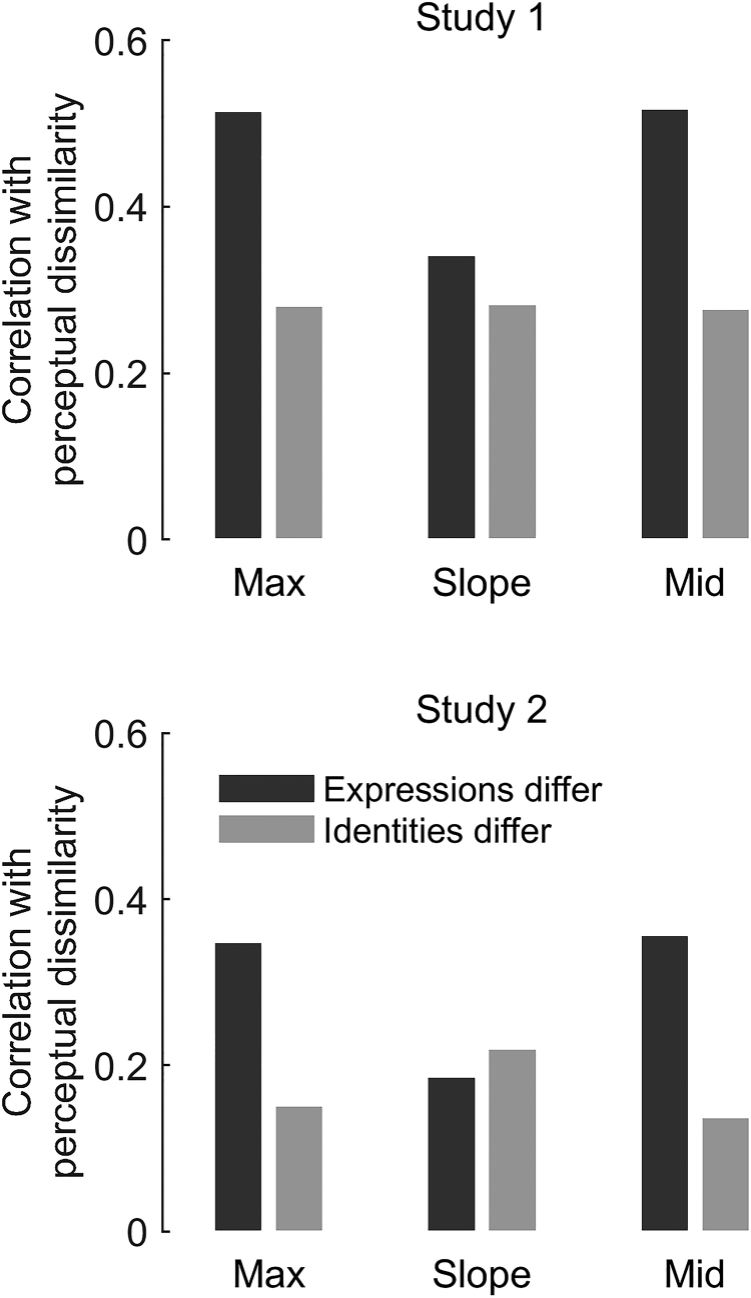
Representational similarity analysis (RSA) of Studies 1 and 2. We assessed if perceived dissimilarity reflected physical motion metrics. We computed correlation coefficients (*y*-axes) between each motion metric matrix (*x*-axis labels) and the perceptual dissimilarity matrices where expressions differ (dark grey bars) or identities differ (light grey bars). See matrices in Figure 2. Perceived dissimilarity better reflects ground truth for expression than for identity.

We also implemented a relatively novel analytic approach to Studies 1 and 2, RSA (Nili et al., 2017), to bolster our conclusions about whether participants’ perception is receptive to the ground truth distinctiveness in the physical stimuli. This statistical procedure, commonly applied to brain imaging data, tests whether there is commonality in the dissimilarity structures (i.e., “representational geometry”) of two dissimilarity matrices by simply correlating them. Here, we used RSA to test the degree to which participants’ perceived dissimilarity matched the ground truth physical dissimilarity in the motion metrics. We found in both Study 1 (Figure 7, top) and Study 2 (Figure 7, bottom) that participants’ perceived dissimilarities of different expressions were better aligned (had higher Pearson's *r*) with motion metrics max and mid than their perceived dissimilarities of different identities. Effects for slope were smaller and inconsistent across studies. Note that, due to the large sample sizes, all statistical comparisons of *r* were highly significant, even after Bonferroni corrections for three tests (expressions-differ vs. identities-differ conditions for each of three motion metrics) per study.

## Study 3: Methods

The pre-registration document for Study 3 and a link to a demonstration of the study are available at https://osf.io/ft53e/.

### Participants

We preregistered a sample size of 300 participants. Of the 300 participants recruited via the Turkprime platform, 284 completed the experiment. Of these, 261 participants passed our pre-registered criterion of at least 75% accuracy on the attention check task (See Procedures, below) and so were included in data analysis. This remaining sample had a mean age of 38, was 47% male, 52% female, with 1% not reporting either. Ninety-five percent claimed English as a native language. Sixty-two percent reported normal vision, 37% reported corrected-to-normal vision and <1% reported unclear yet uncorrected vision. Participants were eligible to participate if they were from the United States and had 95% approval ratings on the platform based on at least 100 tasks. Ethics protocols were approved by the Royal Holloway, University of London College Ethics Board.

### Stimuli

Stimuli included four female identities and four male identities, with caricatured, anticaricatured, and veridical videos of each identity expressing anger, disgust, happy, fear, and surprise expressions and their corresponding antiexpressions.

#### Procedures

Each participant studied two randomly selected male and two randomly selected female identities. One of these male faces and one of the female faces were always assigned to be veridical, while the remaining male and female faces were assigned to be one caricature and one anticaricature, with the two caricature levels randomly assigned to the two genders. All the faces in a given participant's study phase were presented with the same randomly assigned expression or anti-expression. Participants were informed that they would need to remember the faces after study. They also rated each face for attractiveness on a nine-point scale using the keyboard keys 1–9. On each trial, a face video would appear for 2 s (one full cycle of the video) above a prompt to rate attractiveness. After video offset, the prompt would remain on screen until the participants input an attractiveness rating. The same video of each identity appeared twice, interspersed throughout a randomised sequence. The Spearman's correlation between attractiveness ratings for the two identical videos was 0.70, *p* < .001. To encourage and check for attention during study, four trials with distractor videos (with heavily pixelated frames) were randomly interspersed among the eight study videos. Participants were instructed to press the space-bar upon appearance of any of these distractors.

Test trials began following an instruction screen. Test faces included, firstly, two veridical “old” faces, which had been previously studied in their caricatured or anticaricatured versions. Performance on these test faces (i.e., their hit rate) was used to test for a generalisation effect (Table 1). Test faces included, secondly, two old identities that were studied in their veridical versions, but at test one was randomly assigned to be a caricature and the other to be an anticaricature. Performance on these test faces (i.e., their hit rate) was used to test for a superportrait effect (Table 1). Lastly, test faces included two “new” caricatures (one male and one female identity) and two new anticaricatures (one male and one female identity). We used errors on these new test faces (i.e., false alarms) to supplement our hit rate measure when testing the superportrait effect (Table 1). All old test faces challenged participants to recognise different videos (caricature levels) than were shown at study. Nevertheless, the expression or antiexpression of test faces was the same as at study. These test faces were presented in a random sequence.

On each test trial, the test video appeared above the prompt “Did you see this person in stage 1?” and radio buttons for Yes and No. After responding Yes or No, a three-point scale then appeared with options: maybe yes/no, probably yes/no and definitely yes/no. Participants selected an option by adjusting a slider (with invisible initial position) using the mouse. The video was presented either until a response was entered or, if a response was not made before video offset, for eight seconds (four cycles of the video) before it disappeared. After test, participants were asked some demographic and confirmatory questions about age, gender, native language, visual acuity, and anomalous events during the data collection (see Participants section, above).

## Study 3: Results

Our results centre on a number of pre-registered hypotheses as well as supplementary exploratory analyses. Please note that our pre-registered hypotheses, in which we assert positive effects of caricature on recognition of facial identities, were formulated and registered on the basis of previous literature and before the null results for perception of different identities found in Studies 1 and 2 were known.

**Figure 8.**
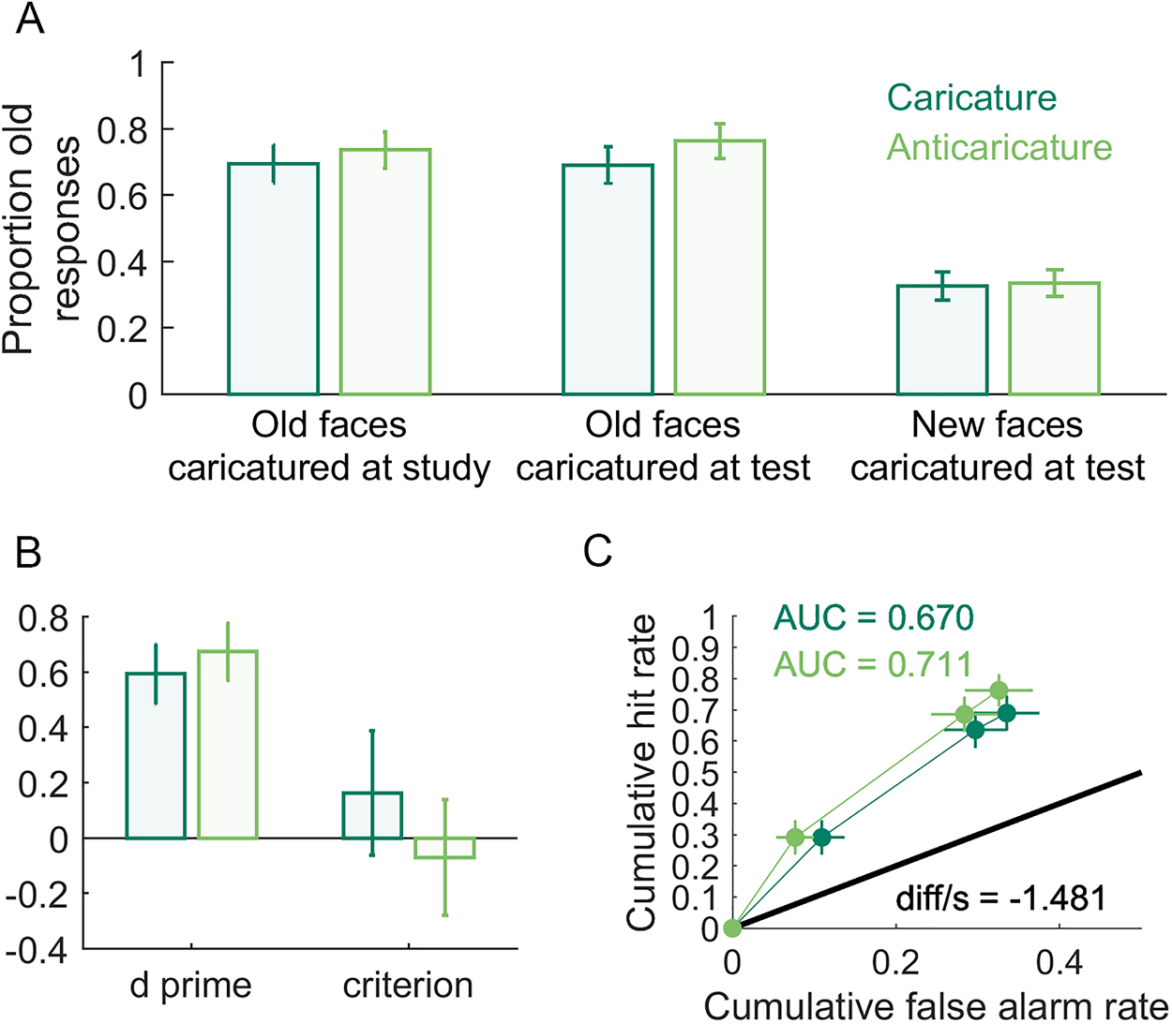
Caricature effects on identity recognition. In (A) are proportion old responses to: (left) faces studied as caricature or anticaricature but tested as veridical (hit rate); (centre) faces studied as veridical but tested as caricature or anticaricature (hit rate); (right) new test faces that were caricatures or anticaricatures (false alarm rate). In (B) are *d′* and *c* for all caricatured and anticaricatured test faces. In (C) are the receiver operating characteristic (ROC) curves and areas under these curves (AUC) for caricatured and anticaricatured test faces. *diff/s* is the difference between the caricatured and anticaricatured AUCs, divided by a bootstrapped estimate of standard error using 10,000.

Our first preregistered hypothesis asserted that caricature of study faces will increase the recognisability of veridical test faces (i.e., a generalisation effect, Table 1). However, in Figure 8A (left bars), anticaricatures appear to have a slightly higher hit rate (proportion old responses to studied faces) than caricatures. Our primary confirmatory analysis to test this hypothesis was, as we pre-registered, a generalised linear mixed model with a binomial logit link function in JASP using stimulus identity and participant as random effects. The fixed effect of study caricature in this analysis was found to be non-significant *χ*^2^(1) = 0.72, *p* = .38. Note that this mixed-effects model attempts to explain whether or not a hit occurred on each trial, and so operates on the data at the individual trial level. The model also simultaneously treats as random effects both variability in participant and facial identity (i.e., items). As exploratory analyses, for this and the dependent variables that follow, we additionally report traditional summary statistics analyses (Table 2), in which performance for trials at each caricature level is averaged either for each participant (participant-level analyses) or for each item (item-level analyses) and then these averages are tested statistically at a second level that treats participants or items as a random effect. For these analyses, we defined “items” as the 8 identities × 5 expressions = 40 items. We moreover report participant- and item-level analyses in the form of both traditional frequentist and Bayesian tests. With the addition of Bayesian *t*-tests (BayesFactor v2b toolbox for MATLAB), we could assess the evidence for null models, where the means for different caricature levels are equal. For the case of old responses to faces caricatured at study, the only evidence from these tests favoured the null model (Table 2).

**Table 2.** *T*-tests comparing caricatured versus anticaricatured faces.

		*bf_01_* ^a^ (amount of evidence)	Frequentist *t*-test
Old faces caricatured at study	Participant-level	7.79 (Moderate)	*t*(260) = −1.12 *p* = .27
	Item-level	4.12 (Moderate)	*t*(39) = −0.87 *p* = .39
Old faces caricatured at test	Participant-level	2.88 (Anecdotal)	*t*(260) = −1.81 *p* = .07
	Item-level	1.15 (Anecdotal)	*t*(39) = −1.9 *p* = .07
New faces caricatured at test	Participant-level	13.59 (Strong)	*t*(260) = −0.35 *p* = .73
	Item-level	5.86 (Moderate)	*t*(39) = 0.01 *p* = .99
*d'*	Participant-level	8.53 (Moderate)	*t*(260) = −1.03 *p* = .30
	Item-level	2.04 (Anecdotal)	*t*(39) = −1.52 *p* = .14
*c*	Participant-level	4.38 (Strong)	*t*(260) = 1.56 *p* = .12
	Item-level	2.33 (Anecdotal)	*t*(39) = 1.42 *p* = .17
AUC	Participant-level	4.92 (Strong)	*t*(260) = 1.48 *p* = .14
	Item-level	0.87 (Inconclusive)	***t*(39) **= **2.06** ***p* **= .05^*^

^a^ Evidence favouring null model.

^*^ *p* *<* *.05*.

Our second preregistered hypothesis asserted that participants would better recognise faces studied in their veridical versions if they are caricatured at test (i.e., superportrait effect). A difference is visible in Figure 8A (middle bars) where the hit rate for anticaricatured faces is slightly higher than for caricatured faces. As with the caricature at study manipulation, this is the opposite direction as we pre-registered. In this case, however, the fixed effect of caricature proved significant in our generalised linear mixed model *χ*^2^(1) = 5.49, *p* = .02. Both participant-level and item-level Bayesian *t*-tests, however, showed anecdotal evidence favouring the null model and the frequentist *t*-tests came just short of significance (*p*s = .07). Note that neither the Bayesian nor frequentist *t*-tests treat hits as discrete outcomes in the way that the binomial link function in the linear mixed model does, nor do the *t*-tests simultaneously model random variations in both stimulus identity (items) and participants, so the results of the mixed model and the *t*-tests need not be identical.

Our third pre-registered hypothesis asserted that participants would more accurately reject caricatured faces as new, resulting in higher false alarm rates (proportion old responses to non-studied faces) for anticaricatured than caricatured identities. Figure 8B (right bars) shows little visible difference in the false alarm rate between caricature conditions. Indeed, there was a null effect in our generalised linear mixed model *χ*^2^(1) < 0.01, *p* = .98 and the evidence from the *t*-tests strongly favours the null models or is non-significant (Table 2).

We also measured the superportrait effect in terms of measures related to signal detection theory: *d′,* criterion (measured as *c*, the distance from the ideal observer threshold), receiver operating characteristic curves (ROC) and the areas under each curve (AUC). We computed *d′* and *c* using the loglinear transform (Stanislaw & Todorov, 1999) to correct for hit and false alarm rates of 0 or 1. We preregistered hypotheses that *d′* and AUC would reflect better sensitivity to studied versus non-studied faces when test faces were caricatured than when they were anticaricatured (Figure 8B). Contrary to *a priori* expectations, the plots of *d′* and the ROC curves shown in Figure 8C if anything visibly suggest some performance advantage for anticaricatures over caricatures. Because these measures are computed as composites over multiple trials, linear mixed models (which operate at the trial level) were not practical. The *t*-tests (Table 2) produced little convincing evidence for differences between caricatures and non-caricatures. However, we note that the item-level analyses for AUC (but not the participant-level analyses) give an inconclusive Bayesian *t*-test result and a significant frequentist *t*-test result. *T*-tests were also consistent with null effects on the criterion measure *c* (Table 2).

## Discussion

Our spatiotemporal caricature technique yielded a stimulus set of unfamiliar faces where Euclidean distances (dissimilarity) between the averaged feature motion patterns (ground truth) of both identity and expression categories were longer for caricatured than for anticaricatured movements. Nevertheless, participants’ dissimilarity perception only reflected this distinctiveness-based “representational geometry” for faces in our stimulus set that differed in expressions but did not for faces that differed in identities. In two studies, participants’ behaviour (for both pairwise rating and multiarrangement paradigms) exhibited greater perceived dissimilarity when expressions differed for pairs of caricatures than pairs of anticaricatures. However, this pattern did not hold for dissimilarity perception of differing identities. Visualisations using MDS showed caricature-related expansion of expression categories in face space but no corresponding expansion for caricatured identities. In a representational similarity analysis, participants’ perceived dissimilarity structure better correlated with the ground truth stimulus dissimilarity structure when expressions differed than when identities differed. In a recognition memory study, little evidence was found for effects of caricature.

In addition to the creation of motion-based caricatures, our study also introduces other novel methods. One novel element is the use of the multiarrangement paradigm used in Study 2. Each participant in Study 2 spatially arranged a pre-designated subset of multiple faces within an iterative series of arenas. This approach contrasts with the traditional method for measuring perception of pairs of stimuli, which involves obtaining ratings from (usually simultaneously-presented) paired combinations of stimuli. Although we did not invent the multiarrangement approach nor its associated inverse MDS algorithm (Kriegeskorte & Mur, 2012; Richie et al., 2020), we here for the first time adapt this approach to the study of face. In doing so, we report a direct and comprehensive comparison of the results from the multiarrangement paradigm with those from the traditional rating method, which validates the multiarrangement approach for investigation of face dissimilarity and/or dynamic stimuli. Indeed, Study 2 successfully replicated Study 1, even though each participant was challenged to arrange a crowded field of faces (at least for the first arena), with all videos playing at once. We conclude from this result that not only are our findings with respect to dissimilarity perception robust, but also that multiarrangement is a viable paradigm for future studies.

Another novel application of a method in the present study involves RSA (Nili et al., 2017) to statistically analyse caricature-related data. Indeed, in Studies 1 and 2, we implemented a mixture of this use of RSA together with more established analyses of participant dissimilarity (e.g., Dawel et al., 2019; Lee et al., 2000) and visualisation using MDS (Johnston et al., 1997; Lee et al., 2000; Nili et al., 2017). RSA additionally can compare the representational geometry of participants’ perceived dissimilarity with that of the physical ground truth. The conclusions from RSA converged with those of Studies 1 and 2: participants’ perceived dissimilarity of faces in our stimulus set shared an overlapping dissimilarity structure with multiple objectively measured motion metrics. However, this representational alignment with physical motion information was more prominent for faces differing in expression, compared to faces differing in identity.

Studies 1 and 2 implemented the experimental approach of measuring perceived dissimilarities in face space to test hypotheses about whether caricature influences recognition of identities and expressions. However, measurement of perceived dissimilarities, like all experimental paradigms, has some limitations. For example, apart from asking participants to try to include motion information in their judgements, participants were otherwise largely free to use whatever information is natural for them as the basis for their dissimilarity perception, leaving some uncertainty about the bases for their judgements. Moreover, we drew conclusions from the results of Studies 1 and 2 about participants’ direct perception of expressions and identities, even though dissimilarity of paired faces is just a proxy measure for direct perception of these categories, and may or may not fully overlap with the visual mechanisms involved. In Furl et al. (2020), we already published a study using an alternative method that measures direct perception of expression categories in our stimulus set. The results of this study agree with those found here in Studies 1 and 2, using dissimilarity paradigms: The degree of caricature successfully improved participants’ expression categorisations. We likewise tested in Study 3 whether caricature of identity-specific information in our face set would produce a more convincing enhancement of behaviour than seen in Studies 1 and 2—but using a very different type of experimental paradigm: one in which participants had to explicitly recognise identity.

This recognition memory study (Study 3) focussed on two types of potential caricature effects (Table 1), which others termed generalisation and superportrait effects (Itz et al., 2017; Kaufmann & Schweinberger, 2012). We designed Study 3 to test for beneficial effects of caricature separately at encoding and test. Although we did not detect either effect, it remains possible that a design where study faces were shown at the same caricature level at both study and test might still yield caricature benefits for the hit rate. Static image caricature effects are most commonly reported using this design (Irons et al., 2014; 2017; Itz et al., 2014; Kaufmann et al., 2013; Kaufmann & Schweinberger, 2012; Schulz et al., 2012; Schultz et al., 2012). Nevertheless, our null findings (reinforced by Bayesian tests of the null model) are still problematic for the idea that recognition memory for identity in the dynamic faces in our stimulus set is based on spatiotemporal face space dimensions. The previously reported findings for static images of generalisation effects for unfamiliar faces (Itz et al., 2017) and superportrait effects for famous and familiar faces (Frowd et al., 2007; Frowd et al., 2012; Lee et al., 2000) and for basic expression categories (Calder et al., 1997; 2000) have usually been interpreted as kinds of superportrait effects. These are accepted as evidence for a face space with dimensions (axes) defined by shape and/or texture information visible in static images. Our results from Study 3 therefore reinforce and broaden the conclusions from Studies 1 and 2: the more distinctive (characteristic, identity-specific) movements of our stimuli may not have been incorporated into representations when processing identity, as they did not benefit identity-related performance in the current studies.

Rather than a superportrait effect, the *less* distinctive *anti*caricatured test faces in fact garnered a *higher* hit rate than caricatured test faces. This finding, if true, would appear to contradict the results of Studies 1 and 2—if participants’ visual systems were simply insensitive to the identity-specific motion information in our stimulus set, then one would predict a null effect for this contrast, rather than an advantage for anticaricatures. Nevertheless, this effect on the hit rate was still rather small, our frequentist mixed model and Bayesian *t*-test disagreed about its presence and it appeared without any corresponding anticaricature advantage on false alarms. Nevertheless, although replication is needed, it is plausible that the anticaricature benefit we observed might reflect a real one, as anticaricature benefits on hit rates were reported in the few static caricature studies that examined superportrait effects using unfamiliar faces (Deffenbacher et al., 2000; Kaufmann & Schweinberger, 2012). Such an anticaricature advantage, if one exists, might be one of many performance differences between familiar and unfamiliar faces. For example, characteristic expressions especially benefit memory for familiar identities (Lander & Butcher, 2020). However, the reasons that participants might make more hits to anticaricatured than caricatured test faces (whether static or dynamic) remains unknown. Perhaps the answer might arise out of recent advances in simulating effects of caricature on identity perception using formal computational models of face space (Hill et al., 2019). Using such methods, future studies can more rigorously characterise how face space representations change as a function of face learning.

Similar work on computational face spaces, such as the analysis of “eigenfaces,” has started to reveal the potential dimensions (axes) that might subserve a purely spatial, image-based face space (Turk & Pentland, 1991). There is less computational work on potential spatiotemporal dimensions. Here, we examined three potential spatiotemporal dimensions of motion that arose naturally from the sigmoidal shape of the motion patterns we observed (Figure 1): the total spatial displacement (max), the speed of the movement (slope), and the timing of the movement (mid). However, the effective dimensionality of the stimulus set was higher than this, as each video's motion was defined on the basis of a pattern over 15 key features, each of which could vary in these three dimensions. In previous dynamic face sets, movements were also implemented as parameterised nonlinear functions over a somewhat limited facial feature space, such as facial action units, a scheme of physiologically inspired muscle displacements (Jack & Schyns, 2015). Using a technique similar to reverse correlation, it can be shown that these stimuli appear to contain sufficient information to explain participants’ expression categorisation performance (Delis et al., 2016). There is also evidence derived from dimensionality reduction techniques on computer-animated faces showing that participants can categorise several facial expressions on the basis of variation along as few as two motion dimensions (Chiovetto et al., 2018). One of our motion metrics was the speed at which the movement took place (the slope of the logistic function). Previous work has shown that participants are sensitive to facial speed when recognising basic expressions (Sowden et al., 2021).

Consistent with the above studies, we find that rendering movements distinctive along spatiotemporal dimensions enhanced dissimilarity perception of faces with different expressions. Moreover, our published work using this stimulus set (Furl et al., 2020) already showed that spatiotemporal caricature successfully enhances accuracy of participants’ expression categorisations. However, unfamiliar face identification is a considerably different and more difficult computational problem than basic expression categorisation. There is much less known that we can use as a guide about the types of motion information that might be diagnostic for individual identities. Participants have already well-learned basic expression categories and so all exemplars from a single expression category (e.g., one individual's smile movement) should resemble to some degree this learned information (e.g., the ends of the mouths will curl upwards), even if a face is unfamiliar. However, encoding identity-specific motion may involve detecting individual variability around prototypic expressions (e.g., one person's smile may curl upwards, but slightly crookedly). Because this latter individualised variability in expression movement will be new to an observer if the face is unfamiliar (as it should have been for our participants), it is difficult to say *a priori* what types of identity-specific information participants’ visual systems might be predisposed to encode (if any).

We used one such formulation—Gaussian variability around the well-learned basic expression prototypes—as our statistical model to generate new identity-specific characteristic movements when we animated our head model. Here, we put this particular hypothetical model of individual movement variability to empirical test. To our knowledge, no study has explicitly tested this model of individual variability. Importantly, our ground truth analysis showed that the type of individual variability we employed is demonstrably amenable to caricature, where the increased distinctiveness can enhance physical dissimilarity between identities, if participants’ visual systems are disposed to detect this enhanced distinctiveness. However, the ground truth analysis also showed that caricature was more effective at enhancing expression information than identity information. It is possible that effects on dissimilarity perception of different identities or on recognition memory of identities might emerge if future iterations of spatiotemporal caricatures stimuli were to caricature the identity-specific information to a much greater degree.

There are several further possibilities for alternate benefits of motion to identity-related perception, which might be tested in future studies. One possibility is that participants’ visual systems simply do not use identity-specific movements when perceiving and recognising unfamiliar faces at all and so participants do not encode such information under normal viewing circumstances. This conclusion at first might seem to conflict with previous findings of motion advantages for processing of unfamiliar face identity (Dobs et al., 2018; Lander & Butcher, 2020). However, others have hypothesised that perception of identity-related differences in dynamic faces could depend predominantly on “structure from motion.” By the structure from motion account, motion information does not provide any direct benefit to identity perception. Instead, motion effectively provides multiple diverse static views of facial positions, affording participants a more comprehensive three dimensional representation of facial form and morphology (Knight & Johnston, 1997; O’Toole et al., 2002). This explanation, while plausible, is beyond the scope of the current study. Our goal here was to measure effects of *motion-based caricature.* From our perspective, any caricature benefit that derives from form, even if motion renders it more visible to participants, would serve as a confound, limiting our ability to draw conclusions about caricature of motion information *per se*. To this end, we attempted to exclude identity-specific form information from the caricature process by mapping facial movements to a single head model that always had the same head shape. Note, our head model did not use the average 2D head shape, which is often used in studies that caricature static form.

Aside from structure from motion, there are still more sources of identity-specific information, which could give rise to motion-based advantages in unfamiliar face identification, and which should be investigated in future studies. Another possibility is that participants are not receptive to differences in identity when they are conveyed by basic expression movements. Ours is not the first study to find limited effects of expression movements on identity-related judgements. Indeed, participants more accurately recognise identity based on movement in the absence of form cues from “conversational” movements than from basic expressions (Dobs et al., 2016).

Yet another possibility is that characteristic movements may require more learning, as they are embedded in identity-expression combinations (e.g., an individual's distinctively slow smile is not visible during the same individual's fast fearful expression movement). In future, an analysis of the cells of the dissimilarity matrix which combine differences in both identities and expressions may yield additional sensitivity to identity-related motion (we did not collect data for these cells, see Methods).

A final possibility is that identity-diagnostic dimensions might rely on abstractions over lower-level motion information, rather than tracking high level feature motion (Giese & Poggio, 2003). In the visual cortex, domain-general motion processing relies on low-level spatiotemporal filter banks like motion energy (Adelson & Bergen, 1985). Just as many models of the ventral pathway of the visual system build higher-level representations of static stimuli out of lower-level filters like Gabor filters (Wang et al., 2016), humans might rely on higher-level motion features abstracted from lower-level ones. In contrast, our landmarking procedure assumed that participants would first recognise high-level shape features (e.g., an eyebrow) and then track their positions. Representations based on tracking the positions of shape features versus motion energy features can lead to differing behaviour (Zaidi & DeBonet, 2000) and there is evidence that biological motion perception might conform to a “dual channel model” that relies on both types of motion representation (Benton et al., 2016; Giese & Poggio, 2003).

Above, we have outlined several different ways in which our computer animations of identity-specific movements might differ from those movements on which the visual system relies when recognising identity. Indeed, our stimuli, because they use a computerised avatar approach, necessarily differ from real faces in many ways. Although our animations relied on several key moveable features of faces (eyebrows, mouth, etc.), they hardly allow the same degrees of freedom in natural human faces. Further empirical data is needed comparing how computer-generated facial motion compares to natural face motion, both in physical stimuli and in how observers perceive them. Nevertheless, we have also described above how facial expressions might be recognisable from relatively few movement dimensions and how participants in our previous study rated these expressions as relatively convincing, compared to the original videos. We view our stimuli as designed to only address the rather specific research question posed here. Thus, we would expect there to be continued development in the realism of computer-animated faces, if computer-animated head models are to have continued use in face recognition research.

We speculate that spatiotemporal face space representations may reside at least in the superior temporal sulcus, if not more widely throughout the visual system. Considerable evidence already has accumulated showing that the superior temporal sulcus, and often other visual areas, are sensitive to facial, body, or biological motion (Atkinson et al., 2012; Foley et al., 2012; Furl et al., 2015; Grosbras et al., 2012; Pitcher et al., 2011; Schultz & Pilz, 2009; Trautmann et al., 2009; Schultz et al., 2013). Now, the field is positioned to move beyond a focus on face-motion-selectivity and to probe the content of facial motion representation and the neural mechanisms that produce them. Some studies have statistically linked motion-based information in faces to neural responses (Deen & Saxe, 2019; Furl et al., 2017; Jabbi et al., 2015) and shown network mechanisms involved in dynamic face motion processing (Furl et al., 2013; Furl et al., 2015). Our group has recently reported suggestive evidence that spatiotemporal face space information is well distributed throughout the visual system (Furl et al., 2020) and may be transmitted in part by beta oscillatory activity (Furl et al., 2017). Our results here, however, raise a new hypothesis that brain responses that subserve spatiotemporal face space dimensions representing identity-diagnostic characteristic movements may be at least partially dissociable from those that subserve expression movements.

In the long term, our results could also motivate applied avenues of research or practice. Caricatures can, in theory, be used to enhance recognition performance for identity recognition in applied contexts from passport matching to eyewitness lineups (identity parades). Work has already begun developing caricature techniques to aid those with low-level visual disorders like macular degeneration (Lane et al., 2018; 2019) and to aid those with chronic inabilities (prosopagnosia) and dementia (Kumfor et al., 2011) to recognise identities and/or expressions. Generalisation and superportrait effects are especially relevant for applied contexts, as one may wish to either (a) use caricature to enhance one's encoding of a face (e.g., a photograph of a sought-after fugitive) to better recognise the veridical face in context later or (b) to use caricature to enhance recognition (e.g., in a photographic eyewitness lineup) of a face previous witnessed in its veridical version (e.g., during a crime). Our findings highlight the challenges of these applications.

In summary, we show that representations expressions can be enhanced using facial movement distinctiveness, manipulated experimentally using a spatiotemporal caricature technique. We empirically tested a particular model of how individual differences in expression movements might be distributed but found little evidence that participants’ visual systems were sensitive to caricature of this type of identity-specific motion. We propose that some dimensions of participants’ face space are spatiotemporal in nature and participants may use these at least when they represent and perceive dynamic facial expressions. As caricature of facial motion is a new technique and field of study, we were not able to survey all possible face perception contexts but, by necessity, chose to focus on certain aspects of face perception. Our most obvious choice was to focus on expression movements and the non-rigid facial feature movements that are most associated with them. Future studies would benefit from studying other types of facial movements, such as speech movements, rigid head movements, and so on.

## References

[bibr1-03010066221086452] Adelson E. H. Bergen J. R. (1985). Spatiotemporal energy models for the perception of motion. Journal of the Optical Society of America A, 2, 284–299. 10.1364/JOSAA.2.000284 3973762

[bibr2-03010066221086452] Ashby F. G. Townsend J. T. (1986). Varieties of perceptual independence. Psychological Review, 93, 154–179. 10.1037/0033-295X.93.2.154 3714926

[bibr3-03010066221086452] Atkinson A. P. Vuong Q. C. Smithson H. E. (2012). Modulation of the face- and body-selective visual regions by the motion and emotion of point-light face and body stimuli. Neuroimage, 59, 1700–1712. 10.1016/j.neuroimage.2011.08.073 21924368

[bibr4-03010066221086452] Bennetts R. J. Butcher N. Lander K. Bate S. (2015). Movement cues aid face recognition in developmental prosopagnosia. Neuropsychology, 29, 855–860. 10.1037/neu0000187 25822463

[bibr5-03010066221086452] Benson P. J. Campbell R. Harris T. Frank M. G. Tovée M. J. (1999). Enhancing images of facial expressions. Perception & Psychophysics, 61, 259–274. 10.3758/BF03206887 10089760

[bibr6-03010066221086452] Benson P. J. Perrett D. I. (1991). Perception and recognition of photographic quality facial caricatures: Implications for the recognition of natural images. European Journal of Psychology, 3, 105–135. 10.1080/09541449108406222

[bibr7-03010066221086452] Benton C. P. Thirkettle M. J. Scott-Samuel N. E. (2016). Biological movement and the encoding of its motion and orientation. Scientific Reports, 22393. 10.1038/srep22393 26925870 PMC4772625

[bibr8-03010066221086452] Blanz V. O’Toole A. J. Vetter T. Wild H. (2000). On the other side of the mean: The perception of dissimilarity in human faces. Perception, 29, 885–891. 10.1068/p2851 11145081

[bibr9-03010066221086452] Brennan S. E. (1985). The caricature generator. Leonardo, 18, 170–178. 10.2307/1578048

[bibr10-03010066221086452] Burton A. M. Vokey J. R. (1998). The face-space typicality paradox: Understanding the face-space metaphor. Quarterly Journal of Experimental Psychology, 51, 475–483. 10.1080/713755768

[bibr11-03010066221086452] Butcher N. Lander K. Fang H. Costen N. (2011). The effect of motion at encoding and retrieval for same- and other-race face recognition. British Journal of Psychology, 102, 931–942. 10.1111/j.2044-8295.2011.02060.x 21988393

[bibr12-03010066221086452] Calder A. J. Rowland D. Young A. W. Nimmo-Smith I. Keane J. Perrett D. I. (2000). Caricaturing facial expressions. Cognition, 76, 105–146. 10.1016/S0010-0277(00)00074-3 10856740

[bibr13-03010066221086452] Calder A. J. Young A. W. Rowland D. Perrett D. I. (1997). Computer-enhanced emotion in facial expressions. Proceedings of the Royal Society of London B: Biological Sciences, 264, 919–925. 10.1098/rspb.1997.0127 PMC1688436 9265191

[bibr14-03010066221086452] Chen J. Tiddeman B. P. (2010). Multi-cue facial feature detection and tracking under various illuminations. International Journal of Robotics and Automation, 25, 162–171. 10.2316/Journal.206.2010.2.206-3386

[bibr15-03010066221086452] Chiovetto E. Curio C. Endres D. Giese M. (2018). Perceptual integration of kinematic components in the recognition of emotional facial expressions. Journal of Vision, 13, 1–19. 10.1167/18.4.13 29710303

[bibr16-03010066221086452] Dawel A. Wong T. Y. McMorrow J. Ivanovici C. He X. Barnes N. Irons J. Gradden T. Robbins R. Goodhew S. C. Lane J. McKone E . (2019). Caricaturing as a general method to improve poor face recognition: Evidence from low-resolution images, other-race faces, and older adults. Journal of Experimental Psychology: Applied, 25(2), 256–279. 10.1037/xap0000180 30321022

[bibr17-03010066221086452] De Leeuw J. R. (2015). Jspsych: A JavaScript library for creating behavioral experiments in a Web browser. Behavior Research Methods, 47, 1–12. 10.3758/s13428-014-0458-y 24683129

[bibr18-03010066221086452] Deen B. Saxe R. (2019). Parts-based representations of perceived face movements in the superior temporal sulcus. Human Brain Mapping, 40, 2499–2510. 10.1002/hbm.24540 30761664 PMC6865455

[bibr19-03010066221086452] Deffenbacher K. A. Johanson J. Vetter T. O’Toole A. J. (2000). The face typicality-recognizability relationship: Encoding or retrieval locus? Memory & Cognition, 28, 1173–1182. 10.3758/BF03211818 11185768

[bibr20-03010066221086452] Delis I. Chen C. Jack R. E. Garrod O. G. B. Panzeri S. Schyns P. (2016). Space-by-time manifold representation of dynamic facial expressions for emotion categorization. Journal of Vision, 16, 14. 10.1167/16.8.14 PMC4927208 27305521

[bibr21-03010066221086452] Dobs K. Bülthoff I. Breidt M. Vuong Q.C. Curio C. Schultz J . (2014). Quantifying human sensitivity to spatio-temporal information in dynamic faces. Vision Research, 100, 78–87. 10.1016/j.visres.2014.04.009 24784699

[bibr22-03010066221086452] Dobs K. Bültoff I. Schultz J. (2016). Identity information content depends on the type of facial movement. Scientific Reports, 6, 34301. 10.1038/srep34301 27683087 PMC5041143

[bibr23-03010066221086452] Dobs K. Bültoff I. Schultz J. (2018). Use and usefulness of dynamic face stimuli for face perception studies-a review of behavioral findings and methodology. Frontiers in Psychology, 9, 1355. 10.3389/fpsyg.2018.01355 30123162 PMC6085596

[bibr24-03010066221086452] Dobs K. Ma W. J. Reddy L. (2017). Near-optimal integration of facial form and motion. Scientific Reports, 7, 11002. 10.1038/s41598-017-10885-y 28887554 PMC5591281

[bibr25-03010066221086452] Foley E. Rippon G. Thai N. J. Longe O. Senior C. (2012). Dynamic facial expressions evoke distinct activation in the face perception network: A connectivity analysis study. Journal of Cognitive Neuroscience, 24, 507–520. 10.1162/jocn_a_00120 21861684

[bibr26-03010066221086452] Frowd C. Bruce V. Ross D. McIntyre A. Hancock P. J. B. (2007). An application of caricature: How to improve the recognition of facial composites. Visual Cognition, 15, 954–984. 10.1080/13506280601058951

[bibr27-03010066221086452] Frowd C. Skelton F. Atherton C. Pitchford M. Bruce V. Atkins R. Gannon C. Ross D. Young F. Nelson L. Hepton G. McIntyre A.H. Hancock P. (2012). Understanding the multiframe caricature advantage for recognizing facial composites. Visual Cognition, 20, 1215–1241. 10.1080/13506285.2012.743936

[bibr28-03010066221086452] Furl N. Begum F. Sulik J. Ferrarese F. P. Jans S. Woolley C. (2020). Face space representations of movement. Neuroimage, 212, 116676. 10.1016/j.neuroimage.2020.116676 32112962

[bibr29-03010066221086452] Furl N. Henson R. N. Friston K. J. Calder A. J. (2013). Top-down control of visual responses to fear by the amygdala. Journal of Neuroscience, 33, 17435–17443. 10.1523/JNEUROSCI.2992-13.2013 24174677 PMC6618361

[bibr30-03010066221086452] Furl N. Henson R. N. Friston K. J. Calder A. J. (2015). Network interactions explain sensitivity to dynamic faces in the superior temporal sulcus. Cerebral Cortex, 25, 2876–2882. 10.1093/cercor/bhu083 24770707 PMC4537434

[bibr31-03010066221086452] Furl N. Lohse M. Pizzorni-Ferrarese F. (2017). Low-frequency oscillations employ a general coding of the spatio-temporal similarity of dynamic faces. Neuroimage, 157, 486–499. 10.1016/j.neuroimage.2017.06.023 28619657 PMC6390175

[bibr32-03010066221086452] Giese M. A. Poggio T. (2003). Neural mechanisms for the recognition of biological movements. Nature Reviews Neuroscience, 4, 179–192. 10.1038/nrn1057 12612631

[bibr33-03010066221086452] Girges C. Spender J. O’Brien J. (2015). Categorizing identity from facial motion. Quarterly Journal of Experimental Psychology (Hove), 68, 1832–1843. 10.1080/17470218.2014.993664 25687732

[bibr34-03010066221086452] Goldstone R. (1994). An efficient method for obtaining similarity data. Behavior Research Methods, Instruments and Computers, 26, 381–386. 10.3758/BF03204653

[bibr35-03010066221086452] Grosbras M. H. Beaton S. Eickhoff S. B. (2012). Brain regions involved in human movement perception: A quantitative voxel-based meta analysis. Human Brain Mapping, 33, 431–454. 10.1002/hbm.21222 21391275 PMC6869986

[bibr36-03010066221086452] Hancock P. J. B. Burton A. M. Bruce V. (1996). Face processing: Human perception and principal components analysis. Memory & Cognition, 24, 26–40. 10.3758/BF03197270 8822156

[bibr37-03010066221086452] Hill H. Johnston A. (2001). Categorizing sex and identity from the biological motion of faces. Current Biology, 11, 880–885. 10.1016/s0960-9822(01)00243-3 11516651

[bibr38-03010066221086452] Hill H. C. H. Troje N. F. Johnston A. (2005). Range- and domain-specific exaggeration of facial speech. Journal of Vision, 5, 4. 10.1167/5.10.4 16441186

[bibr39-03010066221086452] Hill M. Q. Parde C. J. Castillo C. D. Colón Y. I. Ranjan R. Chen J. -C Blanz V. O'Toole A. J . (2019). Deep convolutional neural networks in the face of caricature. Nature Machine Intelligence, 1, 522–529. 10.1038/s42256-019-0111-7

[bibr40-03010066221086452] Irons J. McKone E. Dumbleton R. Barnes N. He X. Provis J. Ivanovici C. Kwa A . (2014). A new theoretical approach to improving face recognition in disorders of central vision: face caricaturing. Journal of Vision, 14, 12. 10.1167/14.2.12 24534882

[bibr41-03010066221086452] Irons J. L. Gradden T. Zhang A. He X. Barnes N. Scott A. F. McKone E . (2017). Face identity recognition in simulated prosthetic vision is poorer than previously reported and can be improved by caricaturing. Vision Research, 137, 61–79. 10.1016/j.visres.2017.06.002 28688907

[bibr42-03010066221086452] Itz M. L. Schweinberger S. R. Kaufmann J. M. (2017). Caricature generalization benefits for faces learned with enhanced idiosyncratic shape or texture. Cognitive, Affective and Behavioral Neuroscience, 17, 185–197. 10.3758/s13415-016-0471-y 27718208

[bibr43-03010066221086452] Itz M. L. Schweinberger S. R. Schulz C. Kaufmann J. M. (2014). Neural correlates of facilitations in face learning by selective caricaturing of facial shape or reflectance. NeuroImage, 102, 736–747. https://www. 10.1016/j.neuroimage.2014.08.042 25173417

[bibr44-03010066221086452] Jabbi M. Kohn P. D. Nash T. Ianni A. Coutlee C. Holroyd T. Carver F. W. Chen Q. Cropp B. Kippenhan S. Robinson S. E. Coppola R. Berman K. F. (2015). Convergent BOLD and beta-band activity in superior temporal sulcus and frontolimbic circuitry underpins human emotion cognition. Cerebral Cortex, 25, 1878–1888. 10.1093/cercor/bht427 24464944 PMC4459288

[bibr45-03010066221086452] Jack R. Schyns P. (2015). The human face as a dynamic tool for social communication. Current Biology, 25, R621–R634. https://www. 10.1016/j.cub.2015.05.052 26196493

[bibr46-03010066221086452] JASP. (Version 0.14) [Computer software].

[bibr47-03010066221086452] Johnston R. A. Milne A. B. Williams C. Hosie J. (1997). Do distinctive faces come from outer space? An investigation of the status of a multidimensional face-space. Visual Cognition, 4, 59–67. 10.1080/713756748

[bibr48-03010066221086452] Kätsyri J. Sams M. (2008). The effect of dynamics on identifying basic emotions from synthetic and natural faces. International Journal of Human-Computer Studies, 66, 233–242. 10.1016/j.ijhcs.2007.10.001

[bibr49-03010066221086452] Kaufmann J. M. Schultz C. Schweinberger S. R. (2013). High and low performers differ in the use of shape information for face recognition. Neuropsychologia, 51, 1310–1319. 10.1016/j.neuropsychologia.2013.03.015 23562837

[bibr50-03010066221086452] Kaufmann J. M. Schweinberger S. R. (2012). The faces you remember: caricaturing shape facilitates brain processes reflecting the acquisition of new face representations. Biological Psychology, 89, 21–33. 10.1016/j.biopsycho.2011.08.011 21925235

[bibr51-03010066221086452] Knappmeyer B. Thornton I. M. Bülthoff H. H. (2003). The use of facial motion and facial form during the processing of identity. Vision Research, 43, 1921–1936. 10.1016/S0042-6989(03)00236-0 12831755

[bibr96-03010066221086452] Knight B. Johnston A . (1997). The role of movement in Face Recognition. Visual Cognition, 4, 265–273. 10.1080/713756764

[bibr52-03010066221086452] Korolkova O. (2018). The role of temporal inversion in the perception of realistic and morphed dynamic transitions between facial expressions. Vision Research, 143, 42–51. 10.1016/j.visres.2017.10.007 29274357

[bibr53-03010066221086452] Kriegeskorte N. Mur M. (2012). Inverse MDS: Inferring dissimilarity structure from multiple item arrangements. Frontiers in Psychology, 3, 245. 10.3389/fpsyg.2012.00245 22848204 PMC3404552

[bibr54-03010066221086452] Krumhuber E. G. Kappas A. Manstead A. S. R. (2013). Effects of dynamic aspects of facial expressions: A review. Emotion Review, 5, 41–46. 10.1177/1754073912451349

[bibr55-03010066221086452] Kumfor F. Miller L. Lah S. Hsieh S. Savage S. Hodges J. R. Piguet O . (2011).) Are you really angry? The effect of intensity on facial emotion recognition in frontotemporal dementia. Social Neuroscience, 6, 502–514. 10.1080/17470919.2011.620779 21957889

[bibr56-03010066221086452] Lander K. Bruce V. (2003). The role of motion in learning new faces. Visual Cognition, 10, 897–912. 10.1080/13506280344000149

[bibr57-03010066221086452] Lander K. Bruce V. Hill H. (2001). Evaluating the effectiveness of pixelation and blurring on masking the identity of familiar faces. Applied Cognitive Psychology, 15, 101–116. 10.1002/1099-0720(200101/02)15:1&lt;101::AID-ACP697>3.0.CO;2-7

[bibr58-03010066221086452] Lander K. Butcher N . (2020). Recognizing genuine from posed facial expressions: Exploring the role of dynamic information and face familiarity. Frontiers in Psychology, 11, 1378. 10.3389/fpsyg.2020.01378 32719634 PMC7347903

[bibr59-03010066221086452] Lander K. Christie F. Bruce V. (1999). The role of movement in the recognition of famous faces. Memory and Cognition, 27(1999), 974–985. 10.3758/BF03201228 10586574

[bibr60-03010066221086452] Lander K. Chuang L. (2005). Why are moving faces easier to recognize? Visual Cognition, 12, 429–442. 10.1080/13506280444000382

[bibr61-03010066221086452] Lander K. Davies R. (2007). Exploring the role of characteristic motion when learning new faces. Quarterly Journal of Experimental Psychology, 60, 519–526. 10.1080/17470210601117559 17455062

[bibr62-03010066221086452] Lane J. Rohan E. M. F. Sabeti F. Essex R. W. Maddess T. Barnes N. He X. Robbins R. A. Gradden T. McKone E. (2018). Improving face identity perception in age-related macular degeneration via caricaturing. Scientific Reports, 8, 15205. 10.1038/s41598-018-33543-3 30315188 PMC6185956

[bibr63-03010066221086452] Lane J. Robbins R. A. Rohan E. M. F. Crookes K. Essex R. W. Maddess T. Sabeti F. Mazlin J. -E. Irons J. Gradden T. Dawel A. Barnes N. He X. Smithson M. McKone E . (2019). Caricaturing can improve facial expression recognition in low-resolution images and age-related macular degeneration. Journal of Vision, 19, 18. 10.1167/19.6.18 31215978

[bibr64-03010066221086452] Lee K. Byatt G. Rhodes G. (2000). Caricature effects, distinctiveness, and identification: Testing the face-space framework. Psychological Science, 11, 379–385. 10.1111/1467-9280.00274 11228908

[bibr65-03010066221086452] Light L. L. Kayra-Stuart F. Hollander S. (1979). Recognition memory for typical and unusual faces. Journal of Experimental Psychology: Human Learning and Memory, 5, 212–228. 10.1037/0278-7393.5.3.212 528913

[bibr66-03010066221086452] Macmillan N. A. Creelman C. D. (1991). Detection theory: A user's guide. Cambridge University Press.

[bibr67-03010066221086452] McIntyre A. H. Hancock P. J. B. Kittler J. Langton S. R. H. (2013). Improving discrimination and face matching with caricature. Applied Cognitive Psychology, 27, 725–734. 10.1002/acp.2966

[bibr68-03010066221086452] McKone E. Robbins R. A. He X. Barnes N. (2018). Caricaturing faces to improve identity recognition in low vision simulations: How effective is current-generation automatic assignment of landmark points? PLoS One, 13, e0204361. 10.1371/journal.pone.0204361 30286112 PMC6171855

[bibr70-03010066221086452] Nili H. , … (2014). A toolbox for representational similarity analysis. PLoS Computational Biology, 10, e1003553. 10.1371/journal.pcbi.1003553 24743308 PMC3990488

[bibr71-03010066221086452] O’Toole A. J. Castillo C. D. Parde C. J. Hill M. Q. Chellappa R. (2018). Face space representations in deep convolutional neural networks. Trends in Cognitive Sciences, 22, 794–809. 10.1016/j.tics.2018.06.006 30097304

[bibr72-03010066221086452] O’Toole A. J. Roark D. A. Abdi H. (2002). Recognizing moving faces: A psychological and neural synthesis. Trends in Cognitive Sciences, 6, 261–266. 10.1016/S1364-6613(02)01908-3 12039608

[bibr73-03010066221086452] Pilz K. S. Thornton I. M. Bülthoff H. H. (2006). A search advantage for faces learned in motion. Experimental Brain Research, 171, 436–447. 10.1007/s00221-005-0283-8 16331505

[bibr74-03010066221086452] Pitcher D. Dilks D. D. Saxe R. R. Triantafyllou C. Kanwisher N. (2011). Differential selectivity for dynamic versus static information in face selective cortical regions. Neuroimage, 56, 2356–2363. 10.1016/j.neuroimage.2011.03.067 21473921

[bibr75-03010066221086452] Pollick F. E. Hill H. Calder A. Paterson H. (2003). Recognising facial expressions from spatially and temporally modified movements. Perception, 32, 813–826. 10.1068/p3319 12974567

[bibr76-03010066221086452] Posner M. I. Keele S. W. (1968). On the genesis of abstract ideas. Journal of Experimental Psychology, 77, 353–363. 10.1037/h0025953 5665566

[bibr77-03010066221086452] Rhodes G. (1997). Superportraits (1st ed.). Psychology Press. 10.4324/9780203304907

[bibr78-03010066221086452] Richie R. White B. Bhatia S. Hout M. C. (2020). The spatial arrangement method of measuring similarity can capture high-dimensional semantic structures. Behavior Research Methods, 52, 1906–1928. 10.3758/s13428-020-01362-y 32077079

[bibr79-03010066221086452] Rodriguez J. Bortfeld H. Gutierrez-Osuna R. (2008). Reducing the other-race effect through caricatures. *Proceedings of the 8th IEEE International Conference on Automatic Face & Gesture Recognition.* 10.1109/AFGR.2008.4813398.

[bibr97-03010066221086452] Rodriquez J. Gutierrez-Osuna R . (2011). Reverse caricatures effects on three dimensional facial reconstructions. Image and Vision Computing, 29, 129–334. 10.1016/j.imavis.2011.01.002

[bibr80-03010066221086452] Ross D. A. Deroche M. Palmeri T. J. (2014). Not just the norm: exemplar-based models also predict face aftereffects. Psychonomic Bulletin and Review, 21, 47–70. 10.3758/s13423-013-0449-5 23690282 PMC4151123

[bibr81-03010066221086452] Schulz C. Kaufmann J. M. Kurt A. Schweinberger S. R. (2012). Faces forming traces: Neurophysiological correlates of learning naturally distinctive and caricatured faces. NeuroImage, 63, 491–500. 10.1016/j.neuroimage.2012.06.080 22796993

[bibr82-03010066221086452] Schulz C. Kaufmann J. M. Walther L. Schweinberger S. R. (2012). Effects of anticaricaturing vs. caricaturing and their neural correlates elucidate a role of shape for face learning. Neuropsychologia, 50, 2426–2434. 10.1016/j.neuropsychologia.2012.06.013 22750120

[bibr83-03010066221086452] Schultz J. Brockhaus M. Bülthoff H. H. Pilz K. S. (2013). What the human brain likes about facial motion. Cerebral Cortex, 23, 1167–1178. 10.1093/cercor/bhs106 22535907 PMC3615350

[bibr84-03010066221086452] Schultz J. Pilz K. S. (2009). Natural facial motion enhances cortical responses to faces. Experimental Brain Research, 194, 465–475. 10.1007/s00221-009-1721-9 19205678 PMC2755747

[bibr85-03010066221086452] Sowden S. Schuster B. A. Keating C. T. Fraser D. S. Cook J. L . (2021). The role of movement kinematics in facial emotion expression production and recognition. Emotion, 21(5), 1041–1061. 10.1037/emo0000835 33661668 PMC8582590

[bibr86-03010066221086452] Stanislaw H. Todorov N. (1999). Calculation of signal detection theory measures. Behavior Research Methods, Instruments, & Computers, 31(1), 137–149. 10.3758/BF03207704 10495845

[bibr87-03010066221086452] Stevenage S. (1995). Can caricatures really produce distinctiveness effects? British Journal of Psychology, 86, 127–146. 10.1111/j.2044-8295.1995.tb02550.x

[bibr88-03010066221086452] Thornton I. M. Kourtzi Z. (2002). A matching advantage for dynamic human faces. Perception, 31, 113–132. 10.1068/p3300 11922118

[bibr89-03010066221086452] Trautmann S. A. Fehr T. Herrmann M. (2009). Emotions in motion: Dynamic compared to static facial expressions of disgust and happiness reveal more widespread emotion-specific activations. Brain Research, 1284, 100–115. 10.1016/j.brainres.2009.05.075 19501062

[bibr90-03010066221086452] Turk M. Pentland A. (1991). Eigenfaces for recognition. Journal of Cognitive Neuroscience, 3, 71–86. 10.1162/jocn.1991.3.1.71 23964806

[bibr91-03010066221086452] Valentine T. (1991). A unified account of the effects of distinctiveness, inversion, and race in face recognition. Quarterly Journal of Experimental Psychology, 43, 161–204. 10.1080/14640749108400966 1866456

[bibr92-03010066221086452] Valentine T. Lewis M. B. Hills P. J. (2016). Face-space: A unifying concept in face recognition research. Quarterly Journal of Experimental Psychology, 69, 1996–2019. 10.1080/17470218.2014.990392 25427883

[bibr93-03010066221086452] Wang P. Gauthier I. Cottrell G. (2016). Are face and object recognition independent? A neurocomputational modelling exploration. Journal of Cognitive Neuroscience, 28, 558–574. 10.1162/jocn_a_00919 26741802

[bibr94-03010066221086452] Yin L. Chen X. Sun Y. Worm T. Reale M. (2008). A high-resolution 3D dynamic facial expression database. In: *Proceedings of the 8th IEEE International Conference on Automatic Face & Gesture Recognition*. 10.1109/FG13733.2008.

[bibr95-03010066221086452] Zaidi Q. DeBonet S. J. (2000). Motion energy versus position tracking: Spatial, temporal and chromatic parameters. Vision Research, 40, 3613–3635. 10.1016/S0042-6989(00)00201-7 11116165

